# αHSA-proIL-12: A Tumor-Conditional IL-12 Prodrug with Reduced Systemic Toxicity and Potent Antitumor Activity

**DOI:** 10.3390/ijms27177716

**Published:** 2026-08-28

**Authors:** Xi Wang, Liu Yang, Yuhao Hou, Guanyu Wang, Yu Ding, Yu Zhang, Shuyu Wang, Zhangyong Hong

**Affiliations:** 1 State Key Laboratory of Medicinal Chemical Biology, College of Life Sciences, Nankai University, Tianjin 300071, China; 2 Nankai International Advanced Research Institute (ShenzhenFutian), Shenzhen 518045, China

**Keywords:** cytokine, immunotherapy, interleukin-12, half-life, prodrug, immune checkpoint blockade (ICB), surgery

## Abstract

Interleukin-12 (IL-12) potently promotes the recruitment and activation of immune cells within the tumor microenvironment (TME), highlighting its immense potential for cancer therapy. However, its dose-limiting systemic toxicity and short half-life severely hinder its clinical translation. Here, we design a tumor-conditionally activated IL-12 prodrug termed αHSA-proIL-12. This engineered molecule integrates a cleavable albumin-binding nanobody (αHSA) domain to extend the half-life and enhance tumor targeting alongside a masking domain that occludes the IL-12 receptor-binding site to prevent nonspecific activation. This construct undergoes selective cleavage by matrix metalloproteinases (MMPs) that are highly expressed in the TME to release highly penetrant, free, low-molecular-weight IL-12 locally. This tumor-restricted activation process directly facilitates the selective recruitment and activation of tumor-infiltrating lymphocytes (TILs). The intravenous administration of an extremely low dose of 108 pmol per dose of αHSA-proIL-12 completely cleared MC38 tumors without inducing systemic toxicity. The maximum tolerated dose of this molecule exceeded 2 nmol, yielding a remarkably wide therapeutic index >18.5. Mechanistically, αHSA-proIL-12 effectively reprogrammed the immunosuppressive TME by expanding the numbers of effector CD8^+^ T cells and natural killer cells while upregulating IFN-γ expression and significantly reducing the proportion of regulatory T cells. This immunological remodeling has the potential to convert “cold” tumors into “hot” tumors. Notably, this treatment achieved complete regression and durable immunity in a subset of mice with multiple refractory immune-cold tumors, including Panc02 pancreatic cancer, 4T1 triple-negative breast cancer, and B16F10 melanoma, when administered as a monotherapy or in combination with PD-L1 blockade. Furthermore, when combined with surgery, αHSA-proIL-12 effectively prevented the postoperative recurrence and distant metastasis of advanced 4T1 tumors. This modular platform provides a clinically promising strategy to overcome the toxicity bottleneck of cytokines and substantially advances the development of combination cancer immunotherapies.

## 1. Introduction

Interleukin-12 (IL-12) serves as a critical orchestrator of innate and adaptive immunity. It efficiently promotes the proliferation and effector functions of natural killer (NK) cells and CD8^+^ T cells within the tumor microenvironment (TME) and induces interferon-γ (IFN-γ) production. This process remodels the immunosuppressive tumor microenvironment and elicits a Th1-type immune response [1], thereby exerting potent antitumor effects [2] and positioning IL-12 as a highly promising candidate for cancer immunotherapy [2,3]. Despite exhibiting remarkable efficacy in multiple murine models, the clinical application of IL-12 has been severely hindered by dose-limiting toxicity (DLT), including life-threatening capillary leak syndrome and hepatotoxicity driven by systemic IFN-γ storms [4,5]. Furthermore, its short circulatory half-life necessitates frequent administration, which further exacerbates the renal burden and systemic toxicity. Consequently, broadening the therapeutic window of IL-12 has emerged as a central challenge in maximizing its clinical translational potential [6].

Genetic engineering of the IL-12 molecule holds promise for broadening its therapeutic window. For instance, Fallon et al. fused IL-12 with an antibody targeting tumor tissue DNA to construct NHS-IL12 (M9241), which increased its accumulation at the tumor site. In phase I clinical trials, this molecule achieved disease stabilization in 30% of patients with metastatic solid tumors; however, it still induced dose-limiting toxicity and systemic inflammation [7,8]. These adverse reactions primarily originated from the nonspecific binding of the drug to circulating immune cells, along with the autoimmune risks triggered by tissue cross-reactivity arising from antigen dependency [9]. The Nirschl and Mansurov groups conjugated IL-12 with blocking antibodies via tumor-cleavable linkers to construct conditionally activated cytokine prodrugs that would overcome this issue, and this approach effectively reduced the systemic toxicity of IL-12 [10,11]. Building upon this foundation, Fu Yang-Xin’s group further utilized the IL-12 receptor to block the activity of IL-12 and fused the prodrug module to the N-terminus of an antibody Fc domain [12]. The resulting engineered molecule was enzymatically cleaved within tumor tissues to release Fc-fused IL-12, thereby extending the in vivo half-life while reducing toxicity and displaying superior therapeutic efficacy. Further broadening its therapeutic window is expected to increase its clinical translational potential.

Fusing protein drugs with anti-human serum albumin (αHSA) nanobodies represents another robust strategy for extending the half-life [13]. This fusion protein can bind to endogenous albumin and bypass lysosomal degradation through the albumin-mediated FcRn recycling pathway, thereby significantly prolonging the circulating half-life of the drug in vivo [14]. This strategy has been successfully applied to the design of the clinical anti-rheumatoid arthritis drug Ozoralizumab. Compared with the Fc fusion strategy, αHSA has a smaller molecular weight (approximately 15 kDa, whereas Fc is approximately 50 kDa), which endows the fusion molecule with an increased capacity to penetrate dense solid tumor tissues [15]. In addition, HSA can accumulate in tumors via gp60-mediated transcytosis and SPARC-mediated retention effects; therefore, it can endow αHSA fusion proteins with a natural tumor tissue-targeting and enrichment ability, which helps to further improve the antitumor therapeutic efficacy of the drug [16,17]. Concurrently, αHSA is immunologically inert [18], thereby avoiding the risks of antibody-dependent cellular cytotoxicity (ADCC) and nonspecific clearance triggered by the interaction of traditional Fc fragments with Fcγ receptors [19,20].

Building on the aforementioned strategies, we designed an αHSA-fused, tumor microenvironment conditionally activated IL-12 prodrug molecule termed αHSA-proIL-12. This molecule fuses αHSA and a masking receptor (IL-12Rβ1) to the N-terminus of IL-12 (a heterodimer of p35 and p40) via a matrix metalloproteinase (MMP)-cleavable linker [21]. It remains in a masked, inactive state during circulation in the blood; upon entering the tumor microenvironment, the cleavage of the linker promotes the dissociation of IL-12 from αHSA and the masking sequence, releasing free, active IL-12 molecules. Our results demonstrate that an extremely low dose of αHSA-proIL-12 (108 pmol/dose) induced complete tumor regression in all MC38 colon cancer-bearing mice, and all the cured mice were completely resistant to tumor rechallenge. The maximum tolerated dose (MTD) of this molecule in mice exceeded 2 nmol/dose, resulting in a therapeutic index (MTD/complete curative dose) of more than 18.5. In multiple refractory immunological “cold tumor” models, including Panc02 pancreatic cancer, 4T1 triple-negative breast cancer, and B16F10 melanoma, monotherapy with αHSA-proIL-12 or its combination with an anti-PD-L1 monoclonal antibody resulted in complete tumor elimination in a subset of mice, with all the cured mice exhibiting complete resistance to homologous tumor rechallenge. In a surgical resection model, the combined administration of αHSA-proIL-12 significantly suppressed the recurrence of advanced 4T1 tumors. Mechanistic studies revealed that αHSA-proIL-12 can effectively remodel the immunosuppressive microenvironment, providing a potent therapeutic strategy for immunological “cold tumors”. In summary, this study provides a promising solution to overcome the toxicity bottleneck of IL-12, suggesting that this drug has significant potential for clinical translation.

## 2. Results

### 2.1. Masked IL-12 Fully Regains Activity upon Treatment with Recombinant Proteases

**Figure 1:**
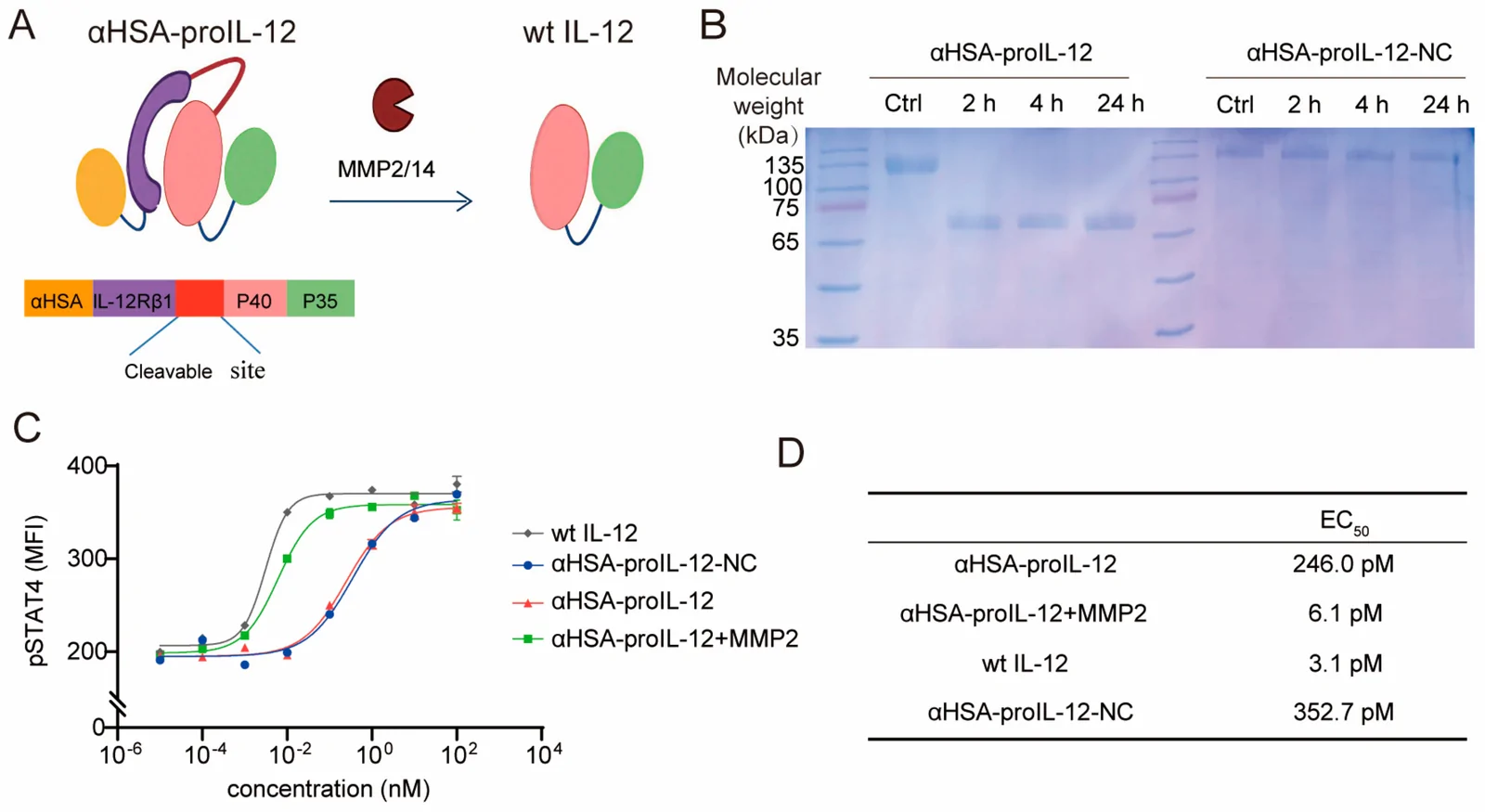
αHSA-proIL-12 fully recovers its activity upon treatment with recombinant proteases. (**A**) Schematic representation of the αHSA-proIL-12 fusion protein architecture. From the N-terminus to the C-terminus, the fusion protein comprises an anti-human serum albumin nanobody (αHSA), an interleukin-12 receptor β1 (IL-12Rβ1) domain, matrix metalloproteinase 2/14 (MMP2/14) recognition sequences, and the p40 and p35 subunits of interleukin-12 (IL-12). Specifically, the αHSA moiety is linked to the subsequent domain via a 3× (G_4_S) flexible peptide linker, while the p40 and p35 subunits are connected by an additional 3× (G_4_S) flexible peptide linker. (**B**) In vitro cleavage of αHSA-proIL-12 and its noncleavable mutant (αHSA-proIL-12-NC) with recombinant MMP2 at 37 °C. After 2, 4, and 24 h, and cleavage products were detected via sodium dodecyl sulfate‒polyacrylamide gel electrophoresis (SDS‒PAGE). (**C**) Dose‒response curves of the levels of phosphorylated STAT4 (p-STAT4) by different IL-12 variants in preactivated primary mouse CD8^+^ T cells (*n* = 3). (**D**) Comparison of EC_50_ values for different IL-12 treatments in inducing STAT4 phosphorylation in CD8^+^ T cells. Abbreviations: αHSA—anti-human serum-albumin nanobody; IL-12—interleukin-12; IL-12Rβ1—interleukin-12 receptor β1; MMP—matrix metalloproteinase; MMP2/14—matrix metalloproteinase-2/-14; NC—non-cleavable mutant; SDS-PAGE—sodium dodecyl sulfate-polyacrylamide gel electrophoresis; p-STAT4—phosphorylated signal transducer and activator of transcription 4; EC_50_—half-maximal effective concentration; MFI—mean fluorescence intensity; wt—wild-type.

We designed a recombinant protein designated αHSA-proIL-12 to generate a tumor conditionally activated form of IL-12 with an extended half-life (Figure 1A). We selected the extracellular domain of IL-12Rβ1 as the masking moiety because of its high affinity for the IL-12 p40 subunit and lower potential for immunogenicity than synthetic peptides or antibodies. This masking domain was fused to an anti-albumin nanobody (αHSA) and tethered to IL-12 via an MMP2/14-sensitive linker (SGQLLGFLTA). Notably, the αHSA domain enables the prodrug to associate with endogenous albumin to significantly prolong its systemic circulation half-life while concurrently facilitating targeted tumor accumulation through the enhanced permeability and retention (EPR) effect and active sequestration by the secreted protein acidic and rich in cysteine (SPARC), which is frequently overexpressed within the tumor stroma. Upon entry into the TME, the linker is cleaved to release free, low-molecular-weight IL-12, thereby ensuring deep tumor penetration and potent local immune activation. A noncleavable mutant (αHSA-proIL-12-NC) and wt IL-12 were generated as controls (Figure S1).

We verified whether αHSA-proIL-12 can be specifically recognized and cleaved by activated MMP2 by incubating αHSA-proIL-12 with active MMP2 (2 ng/μL) at 37 °C for 2 h, 4 h, and 24 h. αHSA-proIL-12 was completely cleaved within 2 h, and the cleaved products remained stable for up to 24 h. In contrast, the noncleavable αHSA-proIL-12-NC mutant retained its intact structure and was not cleaved even after 24 h of incubation. We next evaluated the masking efficiency of αHSA-proIL-12 in vitro. CD8^+^ T cells were treated with various formulations, and the level of STAT4 phosphorylation was assessed using flow cytometry. The ability of αHSA-proIL-12 to induce STAT4 phosphorylation was reduced by approximately 80-fold compared with that of wt IL-12. Following treatment with MMP2, the bioactivity of αHSA-proIL-12 was restored to a level that was comparable to that of wt IL-12 (Figure 1C,D).

### 2.2. αHSA-proIL-12 Has a Wider Therapeutic Window than wt IL-12

**Figure 2:**
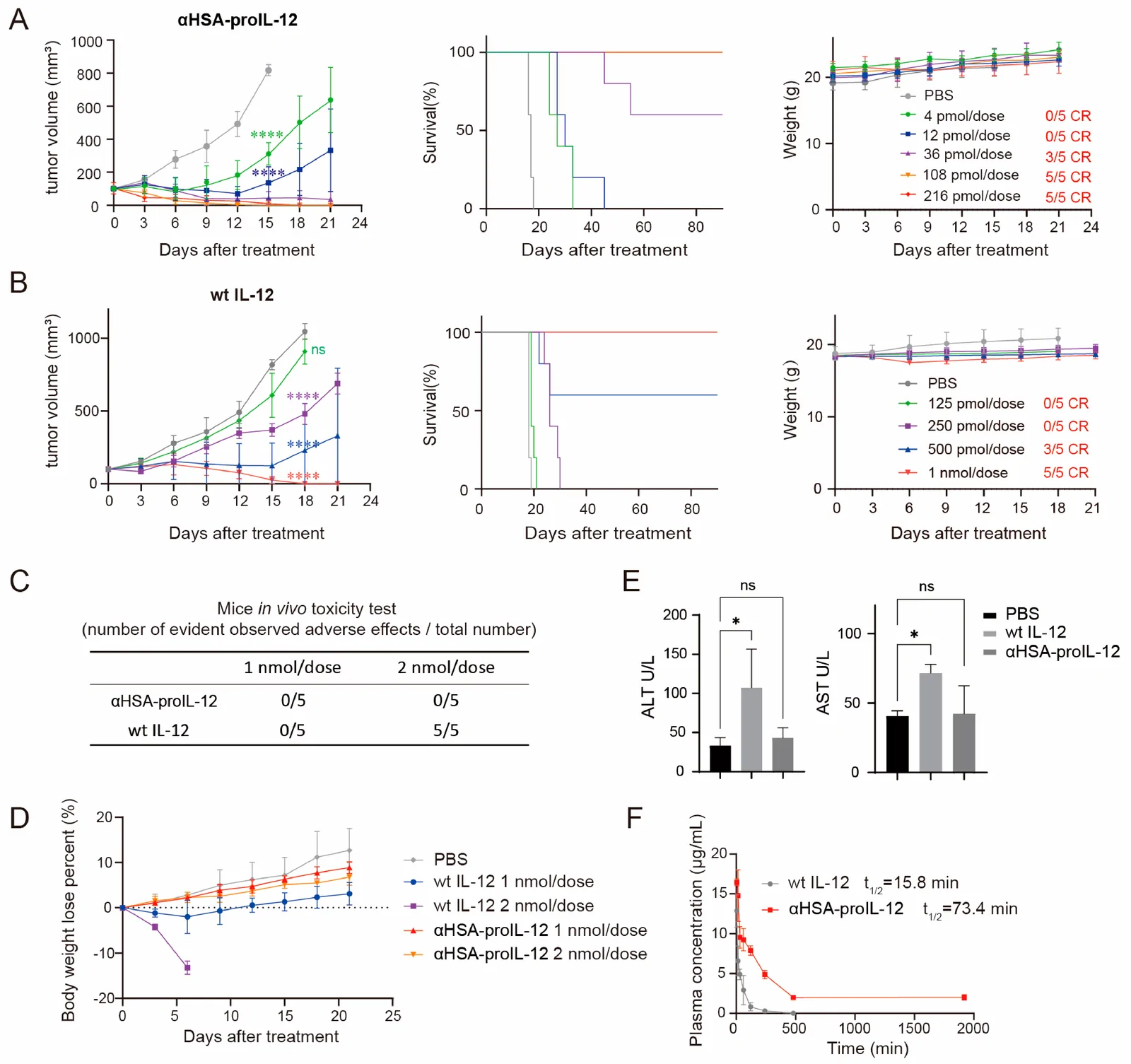
αHSA-proIL-12 exhibited a significantly wider therapeutic window. (**A**,**B**) Antitumor activity of αHSA-proIL-12 and wt IL-12 in MC38 colorectal tumor-bearing mice (*n* = 5). Drugs were intravenously injected (i.v.) every 3 days for 7 cycles at graded doses. (**C**) Proportion of mice presenting overt toxicities (hypoactivity, reduced food intake, >15% body weight loss) after repeated high-dose intravenous treatment for seven doses at 3-day intervals. (**D**) Body-weight changes of MC38 tumor-bearing mice following repeated high-dose administration (*n* = 5); weight was measured every 3 days, with Day 0 defined as the first administration. (**E**) Serum ALT and AST levels in MC38 mice receiving 1 nmol αHSA-proIL-12, 1 nmol wt IL-12 or PBS control. (**F**) Pharmacokinetic profiles of αHSA-proIL-12 and wt IL-12 in Balb/c mice (*n* = 3). Following a single intravenous administration, serum samples were collected at serial time points, and drug half-life (t_1_/_2_) was determined by ELISA. The data are shown as the mean ± SEM. All the data were subjected to one-way analysis of variance (ANOVA), and significant differences between groups (comparisons versus the PBS control group are presented in the figures) were further determined by performing Tukey’s multiple comparison test. * *p* < 0.05, **** *p* < 0.0001, and ns = not significant. Abbreviations: αHSA—anti-human serum-albumin nanobody; wt—wild-type; i.v.—intravenous injection; ALT—alanine aminotransferase; AST—aspartate aminotransferase; PBS—phosphate-buffered saline; ELISA—enzyme-linked immunosorbent assay; SEM—standard error of the mean; ANOVA—analysis of variance; t_1_/_2_—half-life; ns—not significant; CR—complete regression.

We initially evaluated the antitumor efficacy of αHSA-proIL-12 versus wt IL-12 at various dosages in an MC38 colorectal cancer model. Both agents exhibited dose-dependent antitumor activity. However, the effective dose of αHSA-proIL-12 was significantly lower than that of wt IL-12. Within the αHSA-proIL-12 treatment groups, 60% of the mice achieved complete tumor regression at a dose of 36 pmol, while a 108 pmol dose resulted in a 100% cure rate. Notably, αHSA-proIL-12 did not induce significant body weight loss at any of the tested doses (Figure 2A, Table S1). Conversely, wt IL-12 required substantially higher doses, ranging from 500 to 1000 pmol, to achieve comparable antitumor effects (Figure 2B, Table S2).

We further determined the MTD by repeatedly administering high doses to the mice. The proportion of mice that experienced overt toxic side effects, which were defined as decreased activity, reduced appetite, or weight loss exceeding 15% of the initial body weight, is shown in Figure 2C. Following seven administrations every three days, the MTD for wt IL-12 was determined to be between 1 and 2 nmol. In contrast, the MTD for αHSA-proIL-12 exceeded 2 nmol, as no higher doses were tested. Mice treated with 2 nmol of wt IL-12 experienced severe weight loss that necessitated humane euthanasia. In contrast, the body weights of the mice in the αHSA-proIL-12 group remained stable (Figure 2D). In the MC38 therapeutic experiments, αHSA-proIL-12 achieved complete tumor clearance at 108 pmol, whereas the effective dose of wt IL-12 was 500 pmol. These findings collectively demonstrate that αHSA-proIL-12 provides a substantially wider therapeutic window (>18.5) than wt IL-12. Additionally, we collected serum from mice treated with 1 nmol of either αHSA-proIL-12 or wt IL-12 to measure alanine aminotransferase (ALT) and aspartate aminotransferase (AST) levels. The results showed that wt IL-12 treatment led to sharp increases in serum ALT and AST concentrations (Figure 2E, Table S3). In striking contrast, the levels in αHSA-proIL-12-treated mice were not significantly different from those in the PBS control group. Taken together, these results confirm that compared with wt IL-12, αHSA-proIL-12 has the potential to reduce systemic toxicity and broaden the therapeutic window.

### 2.3. αHSA-proIL-12 Exhibits a Prolonged Half-Life In Vivo

We evaluated the pharmacokinetics of αHSA-proIL-12 and wt IL-12 in mice using ELISA. The results indicated that αHSA-proIL-12 had a half-life of approximately 73 min, while that of wt IL-12 was only approximately 15 min (Figure 2F). Throughout the observation period, the circulating concentration of αHSA-proIL-12 remained significantly higher. Cytokines typically exhibit short in vivo half-lives due to their low molecular weight and rapid clearance. This limitation often severely constrains their therapeutic potential. In this study, we incorporated an anti-serum albumin nanobody (αHSA) into the design to extend the circulation time. By binding to serum albumin, HSA effectively sustains elevated plasma levels of IL-12. Given the substantial difference in serum albumin half-life between mice (approximately 1 day) and humans (approximately 19 days), we expect αHSA-proIL-12 to demonstrate superior pharmacokinetic properties in clinical settings. These improvements are likely to further increase its antitumor efficacy.

### 2.4. αHSA-proIL-12 Induces Cleavage-Dependent Tumor Regression and Durable Immunological Memory

**Figure 3:**
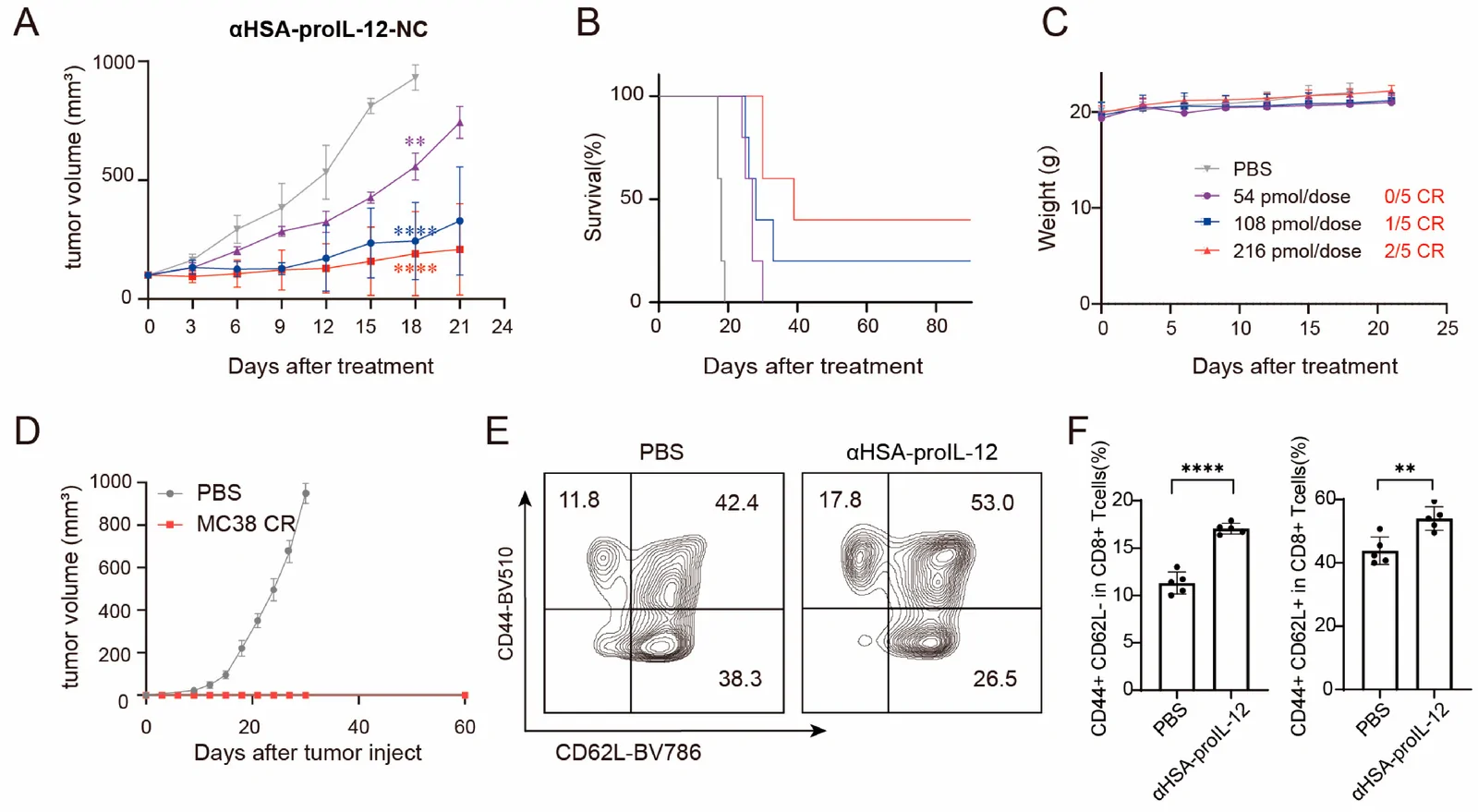
αHSA-proIL-12 induces cleavage-dependent tumor regression and durable immunological memory. (**A**–**C**) Therapeutic efficacy of αHSA-proIL-12-NC in a murine MC38 colorectal cancer model (*n* = 5). Tumor growth curves of mice treated with the indicated regimens (**A**). The data are presented as the mean ± SEM. Kaplan–Meier survival analysis of mice in the indicated groups (**B**). Changes in the body weight of the mice during the treatment period as an indicator of systemic toxicity (**C**). (**D**) Mice that achieved complete tumor regression following αHSA-proIL-12 treatment were subjected to tumor rechallenge by the subcutaneous inoculation of MC38 cells. (**E**) Spleens of cured mice were harvested aseptically at indicated time-points. CD8^+^ T-cell memory markers CD44 and CD62L were detected via flow cytometry. (**F**) The percentages of CD44^+^CD62L^−^ effector memory T cells (TEM) and CD44^+^CD62L^+^ central memory T cells (TCM) were quantified using FlowJov10.8.1 software. The data are shown as the mean ± SEM. One-way ANOVA with Tukey’s multiple-comparison test (**A**) and unpaired two-tailed Student’s *t*-test (**F**) were used for statistical analysis (comparisons versus the PBS control group are presented in the figures). ** *p* < 0.01 and **** *p* < 0.0001. Abbreviations: αHSA—anti-human serum-albumin nanobody; NC—non-cleavable mutant; SEM—standard error of the mean; ANOVA—analysis of variance; TEM—effector-memory T cell; TCM—central-memory T cell; ns—not significant; CR—complete regression.

We investigated the necessity of localized tumor activation for therapeutic efficacy by constructing a noncleavable mutant, αHSA-proIL-12-NC, and evaluated its antitumor activity (Figure 3A–C, Table S4). Notably, the cure rate for αHSA-proIL-12-NC was only 40%, even when administered at high doses (216 pmol). These results indicate that the superior antitumor effects exhibited by αHSA-proIL-12 depend on its MMP2/14-mediated activation within the tumor microenvironment.

We further verified whether αHSA-proIL-12 can induce long-term antitumor immunity by performing a tumor rechallenge experiment through the inoculation of MC38 cells into the contralateral flank of cured mice. None of the mice previously treated with αHSA-proIL-12 exhibited tumor growth (Figure 3D). These observations confirm that αHSA-proIL-12 induces durable antitumor immune memory in vivo. We isolated splenocytes from cured mice and analyzed the memory phenotype of CD8^+^ T cells using flow cytometry to elucidate the underlying mechanism. The results showed that the proportions of both effector memory (CD44^+^CD62L^−^) and central memory (CD44^+^CD62L^+^) CD8^+^ T cells were significantly increased compared with the control group (Figure 3E,F, Tables S4 and S5). Collectively, these findings establish that αHSA-proIL-12 mediates long-term immune protection by promoting the formation of memory T-cell populations.

### 2.5. αHSA-proIL-12 Effectively Eliminates 4T1 Breast Tumors and Markedly Increases the Therapeutic Efficacy of Surgical Resection in a Murine Model

**Figure 4:**
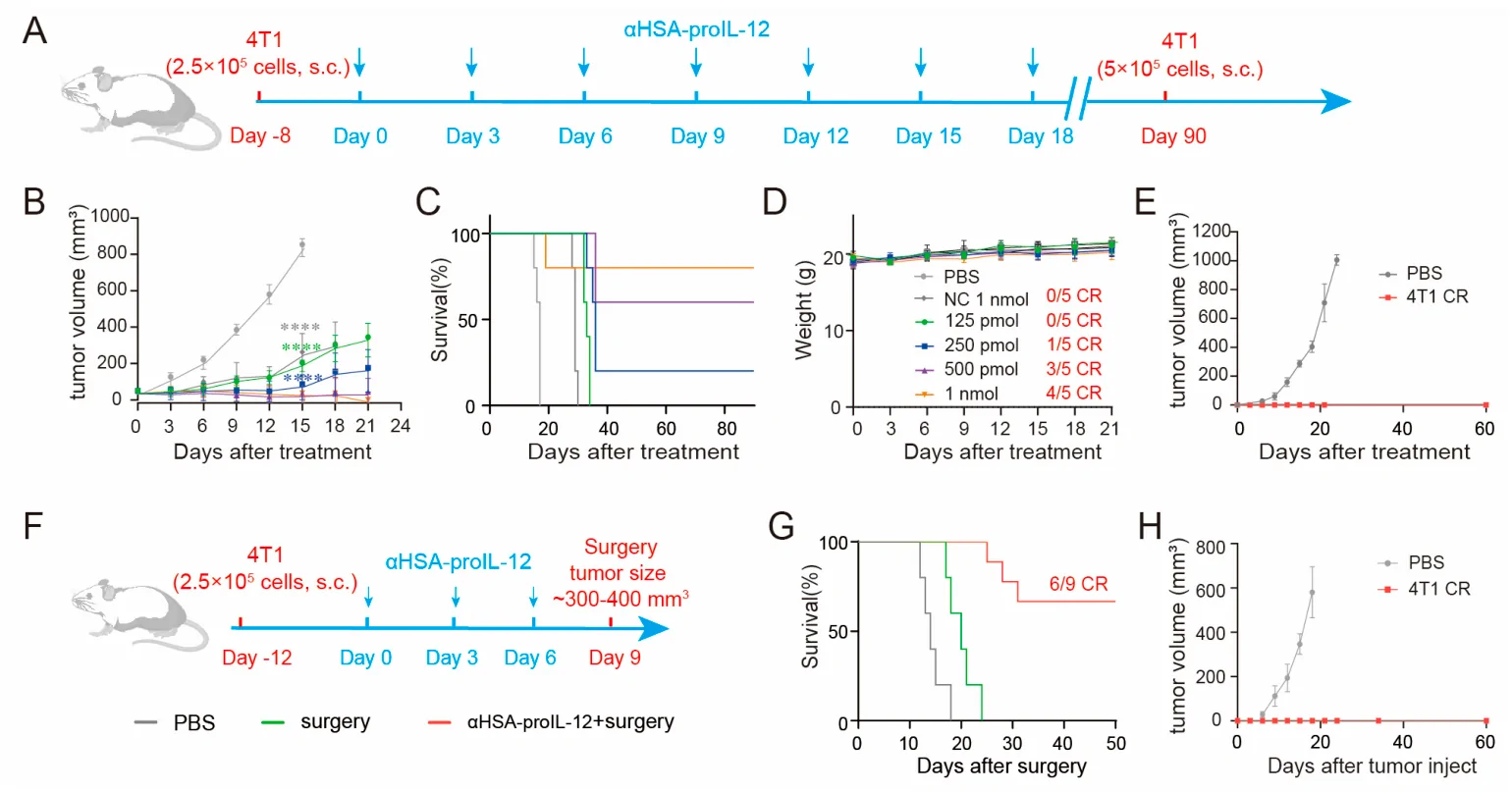
αHSA-proIL-12 can effectively eliminate 4T1 tumors and markedly increase the therapeutic efficacy of surgical resection. (**A**) Therapeutic protocol for the 4T1 murine breast cancer model. At 8 days after tumor cell inoculation, mice whose tumor volumes reached 30–50 mm^3^ were selected randomly for treatment. (**B**–**D**) Mice (*n* = 5) were randomly divided into six groups. Animals were treated with gradient doses of αHSA-proIL-12 (125 pmol, 250 pmol, 500 pmol, and 1 nmol), with the PBS group as the negative control. All treatments were administered via tail vein injection every 3 days. Tumor volume (**B**) and body weight (**D**) were measured every 3 days, and overall survival (**C**) was monitored daily. (**E**) Cured mice from αHSA-proIL-12 treatment underwent tumor rechallenge, and tumor formation was continuously monitored for 60 days. (**F**) Therapeutic protocol for surgical intervention in 4T1 tumor-bearing mice. Mice (*n* = 5) were treated with three doses of αHSA-proIL-12 when the 4T1 tumor volume reached 150–200 mm^3^. Tumor resection was conducted on the 9th day after the initial αHSA-proIL-12 administration. The experimental animals were randomly divided into three groups (*n* = 5–9 mice per group): the PBS control group, the surgery-alone group, and the αHSA-proIL-12 + surgery combined therapy group. (**G**) The survival status of all the mice was monitored and documented daily throughout the entire experimental period. (**H**) A tumor rechallenge assay was performed in mice that were cured after treatment with αHSA-proIL-12, and tumor formation was monitored for 60 days. The data are shown as the mean ± SEM. Statistical significance was determined using one-way analysis of variance (ANOVA) with Tukey’s multiple comparisons test (comparisons versus the PBS control group are presented in the figures). **** *p* < 0.0001.

Next, we evaluated the antitumor efficacy of αHSA-proIL-12 in a subcutaneous 4T1 model. As a well-established immunologically “cold” tumor, the 4T1 model is characterized by a highly immunosuppressive microenvironment, myeloid cell predominance, and T-cell exhaustion. These features render most immunotherapeutic strategies ineffective. In addition, 4T1 tumors exhibit a high metastatic potential. Mice frequently fail to achieve long-term survival due to distant metastasis even after surgical resection of the primary tumor. Consequently, therapeutic intervention in this model remains a substantial challenge. The treatment schedule of αHSA-proIL-12 for mice bearing 4T1 tumors is depicted in Figure 4A. As shown in Figure 4B and Figure S2A, Table S6, αHSA-proIL-12 effectively inhibited tumor growth and induced the regression of early-stage tumors with a volume of 30–50 mm^3^. The cure rate in treated mice reached 60–80%. In contrast, complete regressions were not observed in the group treated with the noncleavable αHSA-proIL-12-NC. However, αHSA-proIL-12 only transiently suppressed the growth of advanced-stage tumors with a volume of 150–200 mm^3^. The tumors in this group regrew rapidly after treatment cessation (Figure S2B).

For advanced 4T1 tumors with an initial volume of 150–200 mm^3^, we further investigated the therapeutic efficacy of αHSA-proIL-12 in combination with surgery as a neoadjuvant regimen. 4T1 tumor-bearing mice with tumor volumes of 150–200 mm^3^ received three doses of 500 pmol αHSA-proIL-12 as pretreatment. The primary tumors were then surgically resected, and the survival of the mice was monitored (Figure 4D). The results showed that mice in the PBS control group rapidly developed tumor recurrence or metastasis after surgery. In comparison, 6 of 9 mice in the αHSA-proIL-12 pretreatment group achieved a complete cure. Only 3 mice developed lung metastasis (Figure 4E). Subsequent tumor rechallenge in these 6 cured mice resulted in no tumor growth, suggesting the induction of potent immune memory (Figure 4F). These findings confirm that αHSA-proIL-12 significantly mitigates the risk of postoperative recurrence and metastasis in late-stage highly metastatic 4T1 tumor models, ultimately increasing the overall surgical cure rate. Although surgical resection remains a foundational standard of care in clinical oncology, the persistent challenges associated with postoperative recurrence and distant metastasis represent a formidable hurdle that limits therapeutic success. Consequently, the development of novel adjuvant strategies centered on αHSA-proIL-12 has substantial potential for clinical translation as a promising approach to improve long-term patient outcomes.

### 2.6. αHSA-proIL-12 Can Effectively Control Immune-Cold Tumors and Exerts a Significant Inhibitory Effect on Tumor Metastasis

**Figure 5:**
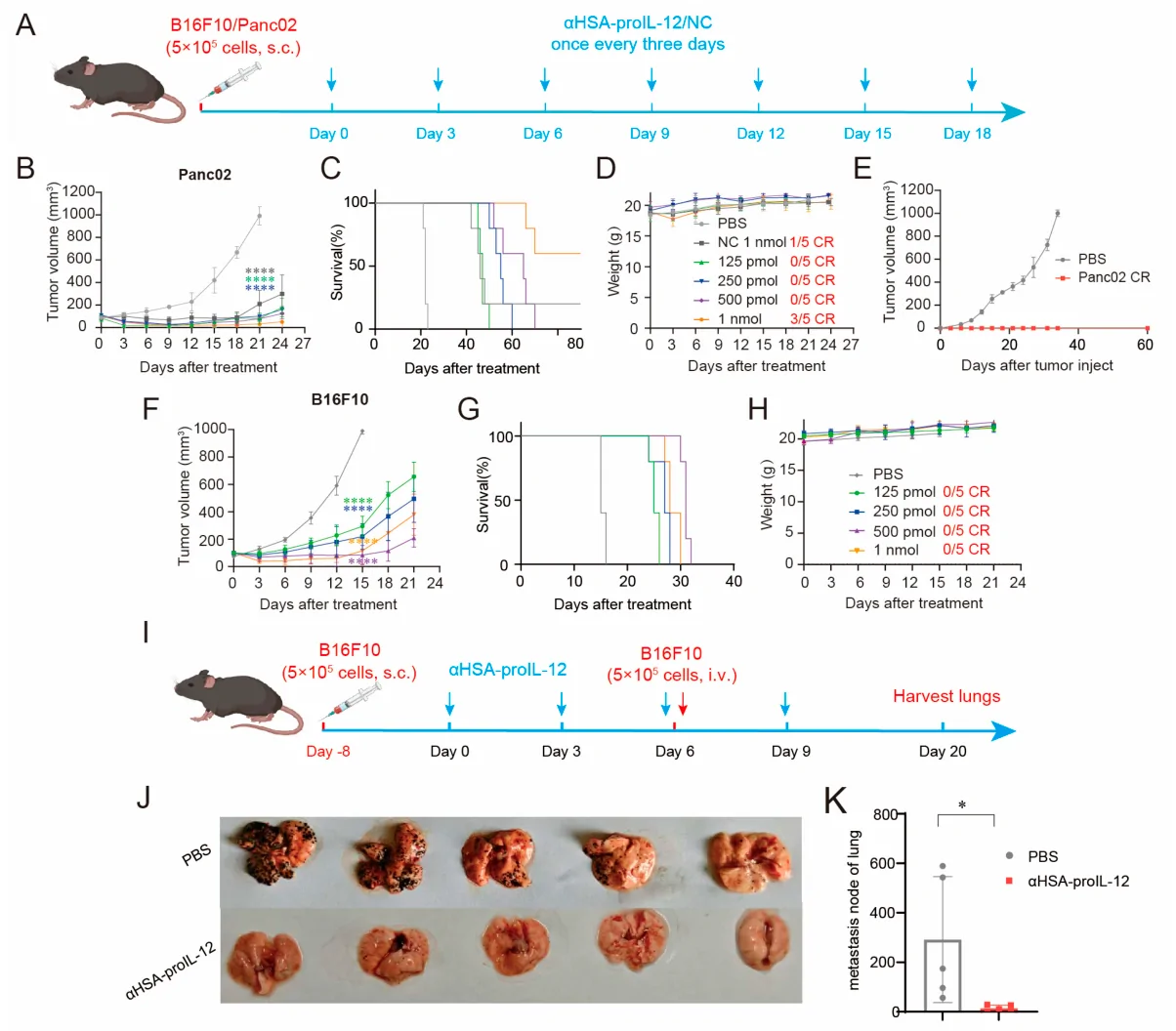
αHSA-proIL-12 can effectively control immunologically cold tumors and significantly inhibit tumor metastasis. (**A**) A total of 5 × 10^5^ Panc02 cells or B16F10 cells were subcutaneously injected into the right dorsal side of each mouse. (**B**–**E**) Mice (*n* = 5) were randomly divided into six groups: the PBS group, the noncleavable protein group (NC), and groups treated with one of four doses of αHSA-proIL-12. The tumor volume (**B**) and body weight (**D**) were recorded every 3 days; the survival time (**C**) of the mice was documented throughout the experiment, and the results observed after tumor rechallenge were also recorded (**E**). (**F**–**H**) The mice (*n* = 5) were randomly divided into five groups: the PBS group and groups treated with one of the four αHSA-proIL-12 doses. The tumor volume (**F**) and body weight (**H**) were recorded every 3 days, and the survival time (**G**) was documented throughout the experiment. (**I**) B16F10 tumor-bearing mice (*n* = 5) were subjected to a tail vein injection of 500 pmol of αHSA-proIL-12 on Days 0, 3, 6, and 9 after tumor cell inoculation. On Day 6, an additional tail vein injection of 5 × 10^5^ B16F10 cells was performed to induce tumor metastasis. On Day 20, the mice were euthanized, and their lungs were aseptically harvested. red arrows represent tumor-cell injection and blue arrows represent drug administration. (**J**) Metastatic lung foci were observed and documented via digital photography**.** (**K**) The number and size of metastatic foci were quantified for subsequent statistical analysis. All results are presented as mean ± SEM. One-way ANOVA with Tukey’s multiple-comparison test (**B**,**F**) and unpaired two-tailed Student’s *t* test (**K**) were used for statistical analysis (comparisons versus the PBS control group are presented in the figures). * *p* < 0.05, **** *p* < 0.0001.

We next evaluated the therapeutic efficacy of αHSA-proIL-12 against Panc02 pancreatic cancer and B16F10 melanoma. Panc02 is a well-established murine pancreatic ductal adenocarcinoma (PDAC) derived from C57BL/6 mice. This tumor recapitulates the clinical hallmarks of PDAC, including poor intratumoral immune cell infiltration and prominent immunosuppressive features. B16F10 melanoma is another well-characterized immunologically “cold” tumor. It shows a poor response to immunotherapy because of the lack of T-cell responses and intratumoral T-cell infiltration. We treated Panc02 and B16F10 tumor-bearing mice with a gradient of doses of αHSA-proIL-12 (Figure 5A). In the Panc02 tumor model, αHSA-proIL-12 exerted a pronounced antitumor effect. It achieved a cure rate of 60% and significantly prolonged the survival of tumor-bearing mice. Furthermore, no tumor growth was observed in the cured mice after rechallenge with the same tumor cells (Figure 5B–E and Figure S3A, Table S7). These observations suggest that these mice developed durable systemic immune memory. In the B16F10 model, αHSA-proIL-12 also exhibited potent growth inhibitory activity, although complete tumor clearance was not observed in this aggressive setting (Figure 5F–H and Figure S3B, Table S8).

We subsequently assessed whether αHSA-proIL-12 could inhibit systemic tumor metastasis. In the experimental metastasis model, mice with established subcutaneous B16F10 tumors received four intravenous doses of αHSA-proIL-12 (500 pmol). We injected B16F10 cells via the tail vein at the time of the third treatment to model the process of metastatic seeding. Two weeks later, we harvested the lung tissues for photographic documentation and quantified the pulmonary metastatic nodules (Figure 5I). The results showed that αHSA-proIL-12 treatment significantly reduced the burden of pulmonary metastasis (Figure 5J,K, Table S9). Notably, two of five mice in the treatment group were entirely free of detectable metastatic nodules. Collectively, these findings indicate that αHSA-proIL-12 has a remarkable ability to drive effective antitumor immunity against immunologically cold tumors.

### 2.7. αHSA-proIL-12 Sensitizes Tumors to PD-L1 Checkpoint Blockade

**Figure 6:**
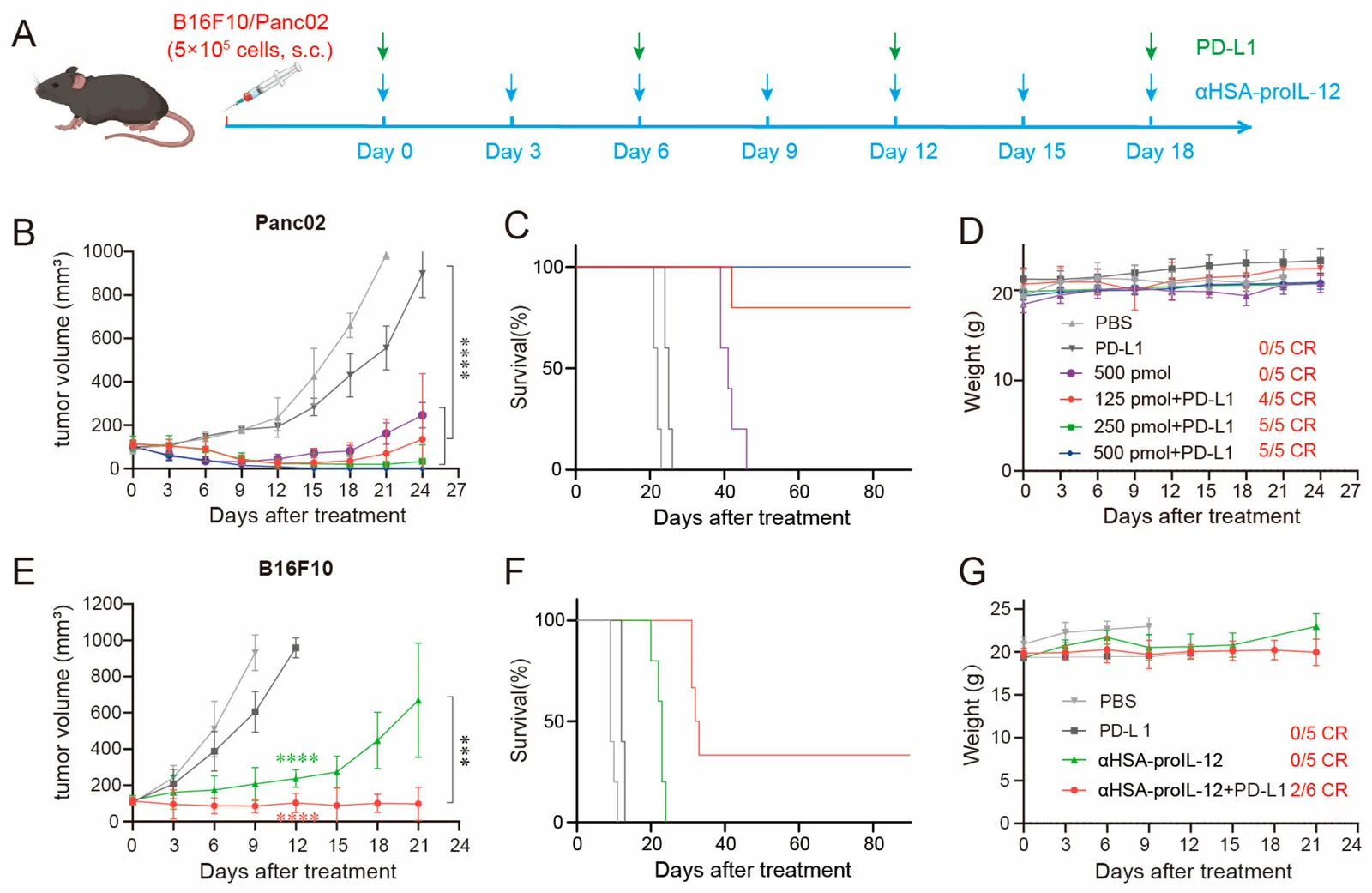
αHSA-proIL-12 significantly enhances the antitumor response to immune checkpoint blockade (ICB) therapy. (**A**) Schedule of the administration of αHSA-proIL-12 combined with the PD-L1 antibody. (**B**–**D**) Evaluation of the antitumor efficacy of αHSA-proIL-12 combined with an anti-PD-L1 antibody in Panc02 tumor-bearing mice. C57BL/6N mice (*n* = 5) were first subcutaneously inoculated with Panc02 pancreatic cancer cells. The mice were randomly assigned to one of the following treatment groups: PBS, anti-PD-L1 antibody alone (200 μg per mouse, i.p. injection every 6 days), αHSA-proIL-12 alone (500 pmol per mouse, i.v. injection every 3 days), and the combination of the anti-PD-L1 antibody (200 μg, i.p. every 6 days) and αHSA-proIL-12 (500 pmol, i.v. every 3 days). The tumor volume (**B**) and body weight (**D**) were monitored every 3 days, and survival was tracked up to 90 days after the initial treatment (**C**). (**E**–**G**) Assessment of the antitumor activity of αHSA-proIL-12 combined with an anti-PD-L1 antibody in B16F10 tumor-bearing mice. C57BL/6N mice (*n* = 5 per group, except for the combination-treatment group with *n* = 6) were subcutaneously implanted with B16F10 melanoma cells. Following the establishment of the tumor and grouping using the same criteria as in the Panc02 model, mice received the corresponding treatments (control, anti-PD-L1 alone, αHSA-proIL-12 alone, or combination therapy) with identical doses and administration schedules. The tumor volume (**E**) and body weight (**G**) were recorded every 3 days, and mouse survival was tracked up to 90 days after the initial treatment (**F**). The data are shown as the means ± SEMs. Statistical significance was determined using one-way analysis of variance (ANOVA) with Tukey’s multiple comparisons test (comparisons versus the PD-L1 control group are presented in the figures; for the B16F10 model, day-21 markers indicate pairwise differences between αHSA-proIL-12 and combination-treatment groups). *** *p* < 0.001 and **** *p* < 0.0001. Abbreviations: ICB—immune-checkpoint blockade; PD-L1—programmed death-ligand 1; i.p.—intraperitoneal injection; i.v.—intravenous injection.

In the B16F10 melanoma and Panc02 pancreatic cancer models, tumors were not completely eradicated in a subset of mice treated with αHSA-proIL-12 monotherapy. Consequently, we attempted to further increase the therapeutic efficacy of the drug against cold tumors through its combination with other treatment strategies. We observed that the tumors initially responded to the treatment but eventually became resistant and resumed growth. We hypothesized that this diminished efficacy in the later stages might be associated with a resurgent immunosuppressive environment. We tested this hypothesis by evaluating whether combining αHSA-proIL-12 with an anti-PD-L1 antibody could elicit increased antitumor activity (Figure 6A).

In the Panc02-tumor bearing mice, the combination of αHSA-proIL-12 and PD-L1 blockade exerted a robust synergistic effect. All five mice in the combination group experienced rapid and sustained tumor shrinkage following treatment initiation. These animals ultimately achieved complete regression (CR) and remained tumor free throughout the 90-day observation period, representing a 100% cure rate (Figure 6B,C, Table S10). Conversely, PD-L1 inhibitor monotherapy had almost no inhibitory effect on tumor growth, with growth curves nearly identical to those of the PBS control group. The survival analysis corroborated these findings, as all the mice in the combination group survived, while those in all the other groups died due to an increased tumor burden within 40 days. Importantly, the body weights of the mice in all the groups remained stable, and no obvious treatment-related toxicity was detected (Figure 6C and Figure S4A).

Consistent with these observations, a marked increase in therapeutic efficacy was observed in the B16F10 melanoma model mice treated with the combination treatment (Figure 6E and Figure S4C, Table S11). Among the six mice that received the combination treatment, two achieved complete tumor clearance (Figure 6F). Although the remaining four mice did not reach complete remission, their tumor growth was significantly restricted compared with that of the control and monotherapy groups. PD-L1 inhibitor monotherapy again failed to effectively control tumor progression. The survival analysis indicated that both median survival and overall survival were significantly prolonged in the combination group. Furthermore, this regimen was tolerated well in the B16F10 model mice, as evidenced by the absence of significant body weight fluctuations (Figure 6G).

These experimental results indicate that the addition of the anti-PD-L1 antibody significantly increases the therapeutic efficacy of αHSA-proIL-12 during tumor treatment. Notably, B16F10 melanoma and Panc02 pancreatic cancer are classified as immunologically cold tumors that typically exhibit a minimal response to PD-L1 monotherapy. The success of this regimen further underscores the potent synergistic effect elicited by the combination of αHSA-proIL-12 and PD-L1 blockade. Consequently, this therapeutic strategy has the potential to substantially increase clinical response rates in patients who remain refractory to current immune checkpoint inhibitors.

### 2.8. αHSA-proIL-12 Reprograms the Tumor Immune Microenvironment

**Figure 7:**
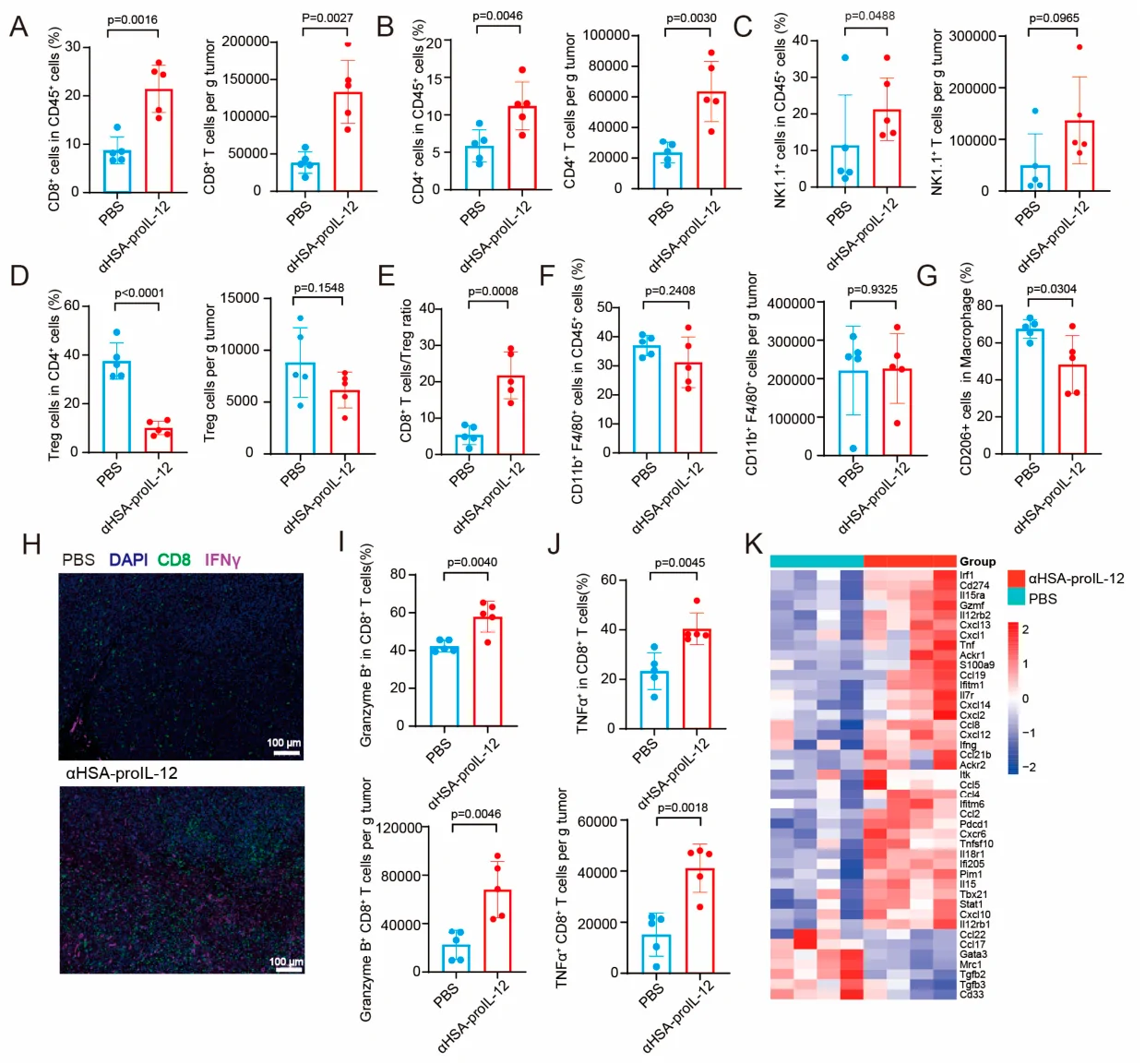
αHSA-proIL-12 treatment increases immune cell activation and infiltration in MC38 tumors. (**A**–**D**) Flow cytometry analysis of tumor-infiltrating immune cell subsets in MC38 tumor-bearing mice after two doses of treatment were administered. The tumors were harvested, and tumor-infiltrating immune cells were isolated via mechanical dissociation and collagenase digestion. The proportions and absolute numbers of (**A**) CD8^+^ T cells, (**B**) CD4^+^ T cells, (**C**) natural killer (NK) cells, and (**D**) regulatory T (Treg) cells (gated as CD4^+^CD25^+^Foxp3^+^) among total tumor-infiltrating immune cells were detected using flow cytometry. (**E**) Ratio of Treg cells to CD8^+^ T cells in MC38 tumors. (**F**,**G**) Analysis of tumor-infiltrating macrophages in MC38 tumors. (**F**) The proportion and absolute number of macrophages (gated on CD11b^+^F4/80^+^) among total tumor-infiltrating immune cells were quantified using flow cytometry. (**G**) The proportion of M2-like macrophages (identified by CD206 expression, gated as CD11b^+^F4/80^+^CD206^+^) among the total macrophage population was further analyzed using flow cytometry. (**H**) Immunofluorescence staining of MC38 tumor sections. After treatment, MC38 tumors were harvested from the mice, fixed with 4% paraformaldehyde, and embedded in paraffin. Tumor sections were stained with DAPI (blue, for nuclear labeling), an anti-mouse CD8 antibody (green, for CD8^+^ T-cell detection), and anti-mouse IFN-γ antibody (purple, for IFN-γ-secreting cell detection). Scale bar = 100 μm. (**I**,**J**) Analysis of the function of tumor-infiltrating CD8^+^ T cells via intracellular cytokine staining. Isolated tumor-infiltrating CD8^+^ T cells were stimulated with phorbol 12-myristate 13-acetate (PMA) and ionomycin (brefeldin A was added to block cytokine secretion) for 4 h in vitro. Flow cytometry was then used to detect (**I**) the proportion and absolute number of CD8^+^ T cells expressing granzyme B and (**J**) the proportion and absolute number of CD8^+^ T cells expressing TNF-α. (**K**) MC38 subcutaneous tumor models were established in C57BL/6N mice. On Day 6 after the initiation of αHSA-proIL-12 treatment, tumor tissues were harvested for a bulk RNA-seq analysis (*n* = 3). Immune-related genes were filtered based on Gene Ontology (GO) annotations and Kyoto Encyclopedia of Genes and Genomes (KEGG) pathway enrichment analysis. Hierarchical clustering was performed, and a heatmap was generated to visualize the differential expression patterns of these immune-related genes between the IL-12-treated and control groups. The data are shown as the means ± SEMs, with statistical significance determined using an unpaired two-tailed Student’s *t* test. Abbreviations: NK—natural killer; Treg—regulatory T cell; DAPI—4′ 6-diamidino-2-phenylindole; IFN-γ—interferon-gamma; PMA—phorbol-12-myristate-13-acetate; TNF-α—tumor-necrosis factor-alpha; RNA-seq—RNA sequencing; GO—Gene Ontology; KEGG—Kyoto Encyclopedia of Genes and Genomes.

We investigated the effects of αHSA-proIL-12 on intratumoral immune cell populations by harvesting MC38 tumors from mice on Day 6 after treatment and isolated immune cells for a flow cytometry analysis. αHSA-proIL-12 treatment significantly increased both the number and proportion of intratumoral CD8^+^ T cells (Figure 7A), CD4^+^ T cells (Figure 7B) and natural killer (NK) cells (Figure 7C). Conversely, the number and proportion of regulatory T (Treg) cells, which constitute an immunosuppressive cell population, were markedly reduced (Figure 7D). These changes led to a significant increase in the ratio of CD8^+^ T cells to Tregs following treatment (Figure 7E), a change that is highly favorable for antitumor immunity in the host. αHSA-proIL-12 treatment slightly decreased the proportion of macrophages in MC38 tumors without altering their total number (Figure 7F). However, the proportion of M2-type macrophages was significantly reduced (Figure 7G), indicating that αHSA-proIL-12 treatment drives the polarization of macrophages from the M2 to the M1 phenotype. A classic biological function of IL-12 is to promote interferon-γ (IFN-γ) secretion by CD8^+^ T cells and NK cells. We validated this result by performing immunofluorescence staining on MC38 tumor tissues. The results revealed significant increases in both the number of CD8^+^ T cells and the intratumoral IFN-γ level following αHSA-proIL-12 treatment (Figure 7H). αHSA-proIL-12 treatment also increased the expression of granzyme B and tumor necrosis factor-α (TNF-α) in intratumoral CD8^+^ T cells (Figure 7I,J), indicating that these cells were in a more activated state.

We further performed an RNA sequencing (RNA-seq) analysis and detected profound transcriptional changes in the tumor microenvironment following αHSA-proIL-12 treatment (Figure S5). We selected immune-related differentially expressed genes to generate a heatmap (Figure 7K). The heatmap showed that the transcript levels of cytokines, chemokines and their receptors associated with immune inflammation were significantly upregulated after treatment. In contrast, the expression of genes that recruit immunosuppressive cells (such as *Ccl17* and *Ccl22*) was markedly downregulated. Genes linked to M2 macrophage polarization (including Mrc1) were also significantly repressed following αHSA-proIL-12 treatment. These transcriptional changes were highly consistent with our flow cytometry findings.

Collectively, these results demonstrate that αHSA-proIL-12 reprograms the MC38 tumor immune microenvironment from an immunosuppressive state to an immunostimulatory phenotype through multiple mechanisms. These mechanisms include promoting the activation and proliferation of effector immune cells, inhibiting immunosuppressive cell populations, and reshaping macrophage polarization.

### 2.9. αHSA-proIL-12 Can Effectively Reverse the Immune Microenvironment of Immunologically “Cold” B16F10 Tumors

**Figure 8:**
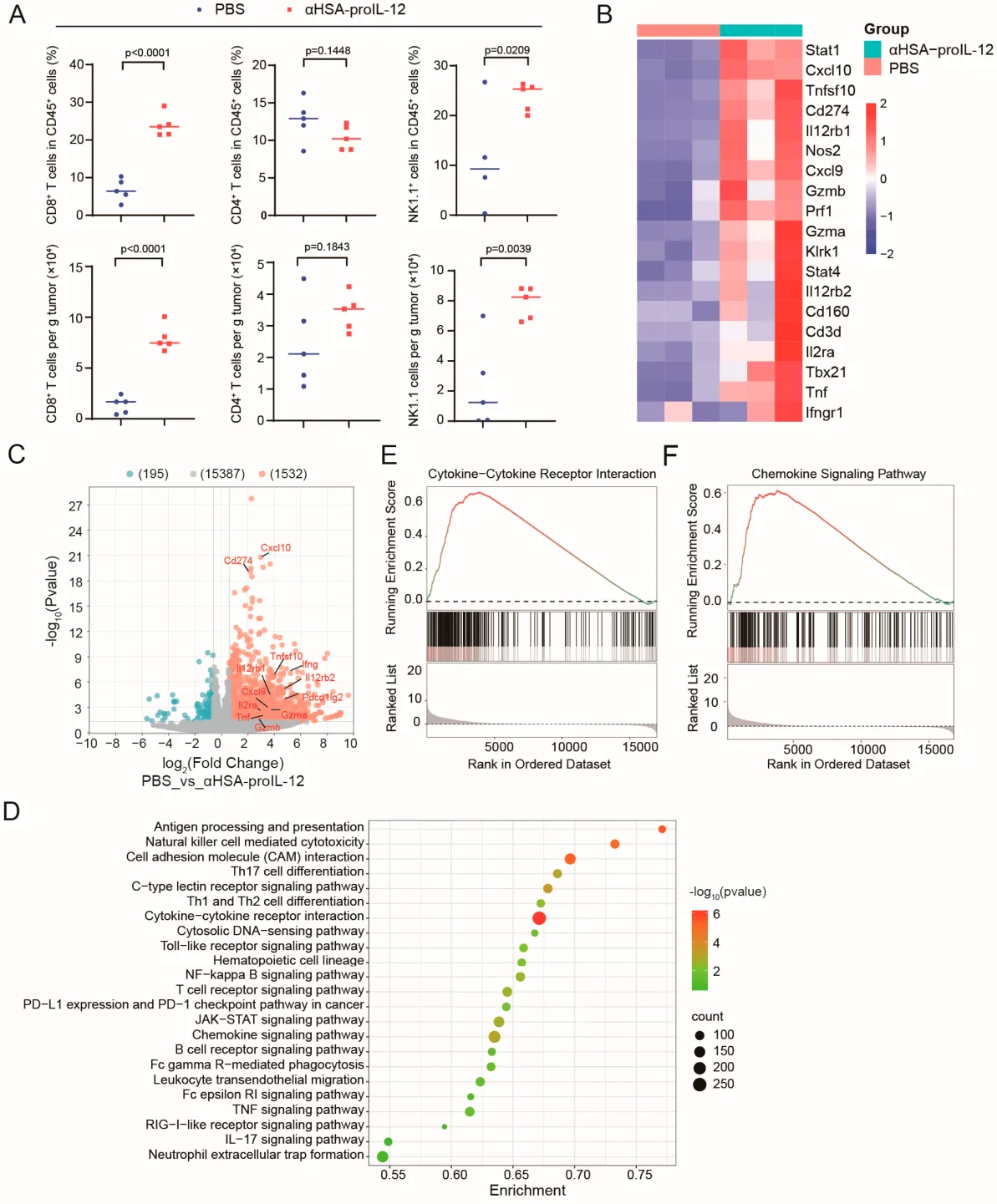
αHSA-proIL-12 can effectively reverse the immune microenvironment of immunologically “cold” B16F10 tumors. (**A**) Flow cytometry-based quantification of the numbers of CD8^+^ T cells, CD4^+^ T cells, and NK1.1^+^ NK cells (as percentages of CD45^+^ immune cells) in B16F10 tumors. The data are shown as the means ± SEMs, with statistical significance determined using an unpaired two-tailed Student’s *t* test. (**B**) Heatmap of the top 20 immune-related genes (hierarchical clustering) in B16F10 tumor tissues from αHSA-proIL-12-treated or PBS control mice. The color intensity indicates the normalized gene expression levels (blue: low; red: high). (**C**) Volcano plot showing differentially expressed genes (DEGs) between αHSA-proIL-12-treated group and the PBS-treated group. Each dot represents one gene; the *x*-axis denotes log_2_(fold change), and the *y*-axis denotes −log_10_(*p* value). Red/green dots indicate significantly upregulated/downregulated DEGs (adjusted *p* < 0.05), and gray dots indicate nonsignificantly altered genes. Key immune-related genes (e.g., *Cd274* and *Ifng*) are labeled. (**D**) KEGG pathway enrichment analysis of the DEGs. The *x*-axis denotes the enrichment score, and the *y*-axis lists the enriched pathways. The dot size indicates the number of genes mapped to each pathway, and the color indicates −log_10_(*p* value). (**E**) GSEA plot of the “cytokine–cytokine–receptor interaction” pathway. The top curve represents the enrichment score (ES), and the black vertical lines indicate the positions of pathway-related genes in the ranked DEG list. (**F**) GSEA plot of the “chemokine signaling pathway”. The plot is interpreted as described in (**E**). Abbreviations: DEGs—differentially expressed genes; KEGG—Kyoto Encyclopedia of Genes and Genomes; GSEA—gene-set enrichment analysis; ES—enrichment score.

We further characterized the intratumoral immune microenvironment of B16F10 cells after treatment using flow cytometry and whole-transcriptome sequencing (RNA-seq) to obtain deeper insights into the differential responses of “cold” and “hot” tumors to αHSA-proIL-12 treatment. Flow cytometry results confirmed that the densities of infiltrating CD8^+^ T cells and NK cells increased significantly in the treatment group, whereas the CD4^+^ T-cell level remained unchanged (Figure 8A). These findings suggest that αHSA-proIL-12 precisely drives the expansion of cytotoxic effector cells within the tumor. The transcriptomic analysis further revealed a profound shift in the intratumoral immune landscape (Figures S6 and S7). Specifically, αHSA-proIL-12 induced the significant upregulation of 1582 genes and the downregulation of 195 genes (Figure 8C). An analysis of immune-related differentially expressed genes revealed that these upregulated genes collectively orchestrated a multidimensional antitumor immune program. This program is driven by four core modules. First, the upregulation of *Il12rb1/2* and *Stat4* confirmed the full activation of the IL-12 signaling axis within the tumor. Second, the synchronized high expression of *Gzma/b*, *Prf1*, *Ifngr1*, and *Stat1* indicated the initiation of a STAT4-IFN-γ-driven effector amplification loop. Third, the increase in *Cxcl9/10* and *Tnf* expression established a proinflammatory chemokine axis that provides the essential momentum for effector cell recruitment. Finally, elevated levels of the NK cell activation receptor *Klrk1* (NKG2D) reflected enhanced innate immune recognition (Figure 8B). KEGG and GSEA enrichment analyses systematically confirmed the construction of this antitumor immune network (Figure 8D–F). The enrichment of the antigen processing and presentation pathway was particularly significant. In combination with activated T-cell receptor signaling and chemokine signaling pathways, these findings indicate that the therapy successfully overcomes the immune tolerance of cold tumors by increasing immunogenicity and directional recruitment. Notably, the intense inflammatory response also induced the compensatory upregulation of *Cd274* (PD-L1) and related pathways. The emergence of this adaptive immune resistance provides a solid theoretical basis for combining αHSA-proIL-12 with PD-1/PD-L1 blockade to achieve durable efficacy.

The aforementioned transcriptomic evidence indicates that αHSA-proIL-12 functions as more than a mere lymphocyte activator in the B16F10 cold tumor model because it can reprogram the immunologically silent microenvironment into a highly proinflammatory state through the remodeling of antigen presentation and chemotactic networks. This comprehensive immune restructuring provides a robust molecular rationale for the potent antitumor responses observed in cold tumors and establishes this therapy as a fundamental component of combination regimens.

A comparative analysis of the transcriptomic profiles between the B16F10 and MC38 models before and after treatment revealed that B16F10 displayed a characteristic cold tumor phenotype in which core genes involved in T-cell infiltration and cytotoxic activity, such as *Cd8a* and *Gzmb*, and MHC molecules, such as *H2-K1* and *Tap2*, were expressed at low levels (Figure S8). Although these genes were upregulated to a limited extent following αHSA-proIL-12 administration, the overall transcript abundance was constrained at a low level. In striking contrast, the MC38 model maintained high baseline expression of these molecules in the absence of treatment, with transcript levels that significantly exceeded those of the treated B16F10 group, and these immune programs were further potently stimulated upon pharmacological intervention. These findings demonstrate that the ceiling of the immune response in B16F10 tumors remains strictly limited by its profound lack of intrinsic antigenicity and low baseline microenvironmental infiltration even upon potent IL-12 signaling. These findings suggest that monotherapy with cytokines is insufficient to overcome the activation threshold of such ultracold tumors, which highlights the need for future strategies that combine cytokine treatment with approaches to enhance antigen presentation or promote immune cell infiltration to achieve superior therapeutic outcomes. All gating strategies for flow-cytometry analysis in this study are provided in Figure S9. Finally, we assessed the antitumor activity of αHSA-prohIL-12, a human IL-12 variant of αHSA-proIL-12, in humanized mice engineered to express human IL-12Rβ1 and IL-12Rβ2, which also exhibited prominent anti-tumor efficacy as shown in Figure S10.

## 3. Discussion

Despite the potent potential of IL-12 to orchestrate antitumor immunity [21,22,23], its clinical translation has been hindered for decades by a persistent “potency–toxicity paradox”. We designed αHSA-proIL-12, a conditionally activated IL-12 prodrug molecule based on an αHSA fusion protein to overcome this bottleneck. This design exploits the intrinsic properties of endogenous albumin to prolong the circulation half-life of the prodrug while facilitating its accumulation within the tumor. The prodrug strategy ensures that the therapeutic agent remains systemically inert before activation at the tumor site, thereby effectively evading peripheral side effects. Benefiting from the relatively small molecular weight of αHSA and the further reduction in size achieved by the complete dissociation of both αHSA and the masking sequence from IL-12 upon activation, this design endows the drug with superior tumor tissue penetration. Our results indicate that αHSA-proIL-12 induces the complete regression of MC38 tumors at concentrations as low as 108 pmol/dose, with a maximum tolerated dose exceeding 2 nmol without significant toxicity, yielding a therapeutic index of more than 18.5-fold. More importantly, this agent alone or in combination with PD-L1 blockade exhibited robust tumor clearance in a subset of mice across multiple refractory immunological “cold” tumor models, including Panc02 pancreatic cancer, 4T1 triple-negative breast cancer, and B16F10 melanoma [24], highlighting its significant clinical translational potential.

Mechanistically, αHSA-proIL-12 treatment recapitulates the classical biological functions of IL-12. In the MC38 tumor model, αHSA-proIL-12 effectively promoted a Th1-type immune response characterized by the infiltration of large numbers of CD8^+^ T cells and NK cells, M1-like macrophage polarization, and a significant increase in intratumoral IFN-γ levels [24,25]. These findings are consistent with those of pioneering studies that established IL-12 as a bridge between innate and adaptive immunity [26,27] and confirm that our masking-release strategy fully preserves the biological potency of the cytokine [10,11]. Through a comparative transcriptomic analysis of “hot” MC38 and “cold” B16F10 models, we found that αHSA-proIL-12 treatment induced an effective Th1 immune response in both tumor types, as evidenced by the significant upregulation of the expression of MHC molecules, chemokines (e.g., *Cxcl9*), and cytotoxic effector molecules [24,28], indicating its therapeutic efficacy against both hot and cold tumors. However, notably, in the B16F10 cold tumor model, the degree of TME activation toward a “hot” phenotype remained far lower than that in the MC38 hot tumor model, even falling below the baseline immunogenicity levels of untreated MC38 tumors. This discrepancy reveals a critical limitation: although IL-12 provides the necessary “ignition” signal, the maximum efficacy is ultimately restricted by the intrinsic immunogenicity of the tumor and the quality of the endogenous T-cell repertoire. Therefore, combination strategies may be required to further increase the activation effect in cold tumors, particularly by increasing the infiltration of immune cells.

Our data further revealed that the potent IFN-γ production induced by αHSA-proIL-12 treatment elicited a compensatory adaptive immune resistance mechanism, which manifested as a marked upregulation of intratumoral PD-L1 expression [29]. This observation is consistent with established reports that IL-12-mediated IFN-γ secretion establishes a negative feedback loop by inducing the expression of inhibitory ligands, such as PD-L1 and indoleamine 2,3-dioxygenase (IDO) [30], in the TME [29,31]. Specifically, studies have shown that while IL-12 activates T-cell effector functions, the concurrent IFN-γ-dependent upregulation of PD-L1 expression can limit the duration of the antitumor response [32]. Based on these results, we combined αHSA-proIL-12 treatment with PD-L1 antibody blockade, which significantly increased the efficacy of the former in multiple tumor models [33], achieving a 100% cure rate in the Panc02 tumor-bearing model mice and complete tumor clearance in 2 of 6 mice bearing B16F10 tumors. These findings indicate that the negative regulation of adaptive immunity in the TME induced by IL-12 can be effectively reversed by combining it with immune checkpoint inhibitors [34]. More importantly, these results suggest that IL-12 not only serves as an effective monotherapy but also holds promise for significantly sensitizing “cold” tumors, which are typically unresponsive to PD-L1 blockade, to immune checkpoint inhibitors.

In addition to serving as a direct therapeutic intervention for established tumors, αHSA-proIL-12 also has notable clinical potential for neoadjuvant and adjuvant applications. In a highly metastatic advanced 4T1 tumor model [35], perioperative administration effectively cleared micrometastases and prevented postoperative recurrence. This finding highlights a major unmet clinical need, as post-operative metastasis of triple-negative breast cancer and other malignancies remains a primary cause of cancer-related mortality. All the cured mice exhibited 100% resistance to tumor rechallenge, underscoring the systemic immune memory induced by αHSA-proIL-12 treatment, which provides a robust safeguard against long-term recurrence through immunosurveillance.

Currently, two other masked IL-12 design strategies have been reported [10,11,12]. One of these uses an anti-IL-12 antibody to block IL-12 activity; however, activation of this molecule requires two cleavage events, which may introduce variability in the activation process [10,11]. The other utilizes an Fc fragment for half-life extension, but the relatively large molecular size of the Fc domain may impede effective penetration of the drug into deep regions of solid tumors [12]. In contrast, our design employs a smaller anti-albumin nanobody to extend circulatory half-life and requires only a single proteolytic cleavage to release fully free IL-12. Owing to its reduced molecular weight, our design facilitates deeper tumor penetration and holds greater potential for robust immune activation within the tumor microenvironment.

However, despite the promising prospects of αHSA-proIL-12, its clinical translation still faces several challenges. First, its activation depends on MMPs, whose expression varies by tumor type, patient population, and stage, suggesting that future clinical trials may require patient stratification strategies based on protease profiling to identify the optimal beneficiary population. Second, the therapeutic efficacy of αHSA-proIL-12 in immune “cold” tumors requires further optimization, as a subset of mice still failed to achieve complete tumor eradication, highlighting the need for rational combination with additional therapeutic strategies to further increase its efficacy. Third, the current investigational data are primarily based on murine models, which differ markedly from humans with respect to the tumor microenvironment, IL-12 signaling mechanisms, and the biological properties of serum albumin. Therefore, parameters obtained from mouse experiments cannot be directly extrapolated to human patients. Nonetheless, the modular nature of this platform allows rapid adaptation to other cytokines, establishing a versatile technical framework for the next generation of precision cytokine-based immunotherapy.

## 4. Materials and Methods

### 4.1. Cell Lines and Animals

The MC38, 4T1, B16F10, and Panc02 cell lines were acquired from the American Type Culture Collection (ATCC, Manassas, VA, USA). All the aforementioned cell lines were cultured in RPMI 1640 medium (Gibco, San Diego, CA, USA) supplemented with 10% fetal bovine serum (FBS; Biological Industries, Kibbutz, Israel) and 1% penicillin‒streptomycin (NCM Biotech, Newport, RI, USA, Cat. #C100C5).

Female C57BL/6N and BALB/c mice (6–8 weeks old) were obtained from Beijing Vital River Laboratory Animal Technology Co., Ltd. (Beijing, China). All animal experiments were approved by the Animal Care and Use Committee of Nankai University (Tianjin, China) prior to initiation.

### 4.2. Production and Characterization of αHSA-proIL-12

αHSA-proIL-12 was designed as a single-chain fusion protein, comprising a nanobody against human serum albumin, murine IL-12Rβ1, a polypeptide sequence cleavable by MMP2 and MMP14 (SGQLLGFLTA), murine p40, and murine p35 in sequence from the N-terminus to the C-terminus. Wild-type IL-12 was constructed by fusing p40 and p35 via a 3 × G4S linker. For the noncleavable variant (αHSA-proIL-12-NC or NC), the cleavable sequence in αHSA-proIL-12 was replaced with a 2 × G4S linker. These proteins were expressed using the Expi293 transient expression system and purified by immobilized metal affinity chromatography via their N-terminal 6 × histidine tag. Briefly, expression plasmids were transfected into 293F cells using polyethyleneimine (PEI). After 7 days of culture, the cellular supernatant was collected and incubated with Ni Sepharose Excel resin for a minimum of 4 h. The resin was harvested using a gravity column, and the target proteins were eluted with imidazole and subsequently dialyzed to remove residual imidazole. Protein purity was characterized by sodium dodecyl sulfate‒polyacrylamide gel electrophoresis (SDS‒PAGE) under reducing conditions. Finally, high-purity proteins were aliquoted and stored at −80 °C until use.

### 4.3. In Vitro Activation of αHSA-proIL-12 with Recombinant Enzymes

Recombinant MMP2 (MCE, HY-148905) was activated prior to its experimental use. MMP2 was diluted to a final concentration of 40 μg/mL in assay buffer (50 mM Tris, 10 mM CaCl_2_, 150 mM NaCl, 0.05% (*w*/*v*) Triton X-100, pH = 7.5), and 4-aminophenylmercuric acetate (APMA, MCE, HY-P73296) was added to achieve a final concentration of 1 mM. The mixture was incubated in a 37 °C water bath for 2 h to induce activation. αHSA-proIL-12 was coincubated with activated MMP2 at a final concentration of 1 μg/mL at 37 °C to verify whether αHSA-proIL-12 could be specifically recognized and cleaved by activated MMP2. Samples were collected at 2, 4, and 24 h and subjected to SDS‒PAGE analysis to assess cleavage efficiency.

### 4.4. Phospho-STAT4 Assay

Purified CD8^+^ T cells (1 × 10^6^ cells/mL) were activated for 3 days in 6-well plates precoated with 5 μg/mL α-CD3 antibody (clone 17A2, BioXcell, Lebanon, NH, USA) supplemented with soluble α-CD28 antibody (5 μg/mL, clone 37.51, BioLegend, San Diego, CA, USA) and murine IL-2 (50 ng/mL, Peprotech, Cranbury, NJ, USA). The culture medium consisted of RPMI 1640 medium (Gibco, Waltham, MA, USA) supplemented with 10% heat-inactivated FBS, 1% penicillin/streptomycin, and 50 μM 2-mercaptoethanol (Sigma‒Aldrich, St. Louis, MO, USA). After 3 days of activation, the CD8^+^ T cells were incubated with fresh culture medium for 6 h and then seeded into 96-well plates at a density of 5 × 10^4^ cells per well. Different concentrations of IL-12 or masked IL-12 (either cleaved or intact) were coincubated with the CD8^+^ T cells at 37 °C for 15 min to induce STAT4 phosphorylation.

Afterwards, the cells were fixed with 4% paraformaldehyde for 10 min at room temperature and then permeabilized with BD Phosflow Perm Buffer III on ice for 30 min. Cells were stained with an AF488-conjugated anti-phospho-STAT4 (Tyr693) antibody (clone 38, BD Biosciences, Milpitas, CA, USA) for 1 h at 4 °C in the dark. The capacity of cytokines to induce STAT4 phosphorylation was quantified by plotting the mean fluorescence intensity (MFI) of the pSTAT4^+^ cell population against the cytokine concentration. Dose‒response curves were fitted using GraphPad Prism software (version 8, GraphPad Software, Inc., Boston, MA, USA).

### 4.5. Safety and Toxicity Assessments

MC38 tumor-bearing mice were administered 1 nmol or 2 nmol of either αHSA-proIL-12 or wt IL-12 via a dosing schedule of one injection every three days for a total of seven doses to determine the MTD and safety profile. During this period, we closely monitored the mice for the appearance of systemic toxic side effects and recorded fluctuations in body weight to assess the overall health status. After 7 doses of 1 nmol αHSA-proIL-12 or wt IL-12 were administered, serum was collected from each mouse, and ALT/AST concentrations were determined using ELISAs.

### 4.6. Pharmacokinetic Analysis

BALB/c mice were administered αHSA-proIL-12 or wt-IL-12 via a tail vein injection at a dose of 2 mg/kg (19.8 nmol/kg for αHSA-proIL-12 and 36.4 nmol/kg for wt IL-12). Blood samples were collected at 5 min, 15 min, 30 min, 1 h, 2 h, 4 h, 8 h, and 24 h after administration, followed by centrifugation to separate the serum. The concentrations of αHSA-proIL-12-NC or wt IL-12 in mouse serum were quantified using ELISA.

### 4.7. Tumor Inoculation and Treatments

For subcutaneous tumor models, 5 × 10^5^ MC38, 5 × 10^5^ B16F10, or 6 × 10^5^ Panc02 cells were injected subcutaneously into the right flank of C57BL/6N mice. For the 4T1 tumor model, 2.5 × 10^5^ 4T1 cells were injected subcutaneously into the right flank of BALB/c mice. The tumor volumes were measured twice weekly using digital calipers, and the volumes were calculated using the following formula: length × width^2^/2.

The mice were assigned to experimental groups via simple stratified randomization. Specifically, following tumor inoculation, the animals were stratified by tumor volume to ensure balanced mean baseline tumor sizes across groups. All tumor volume measurements were performed in a blinded fashion, with operators unaware of group allocation. Predefined exclusion criteria were applied: animals with failed tumor engraftment, those with tumor burden exceeding ethical limits, or those experiencing non-tumor-related accidental death were excluded from subsequent analyses.

For the MC38, Panc02, and B16F10 monotherapy models, the mice were enrolled once tumors reached a uniform volume of 100–150 mm^3^, and then randomly allocated to receive intravenous injections of PBS, αHSA-proIL-12, αHSA-proIL-12-NC, or wild-type IL-12 every 3 days for a total of 7 doses.

For the early-stage 4T1 monotherapy model, the mice were enrolled at tumor volumes of 30–50 mm^3^ and received escalating doses of αHSA-proIL-12, αHSA-proIL-12-NC, wild-type IL-12, or PBS via intravenous injection every 3 days for 7 total doses. Tumor progression, body weight, and overall survival were monitored at regular intervals throughout the study. For the 4T1 neoadjuvant surgical model, the mice bearing primary 4T1 tumors (150–200 mm^3^) were randomly assigned to three groups: PBS control, surgery alone, and αHSA-proIL-12 in combination with surgery. The mice in the combination group received preoperative neoadjuvant therapy consisting of 500 pmol/mouse αHSA-proIL-12 administered via tail-vein injection every 3 days for a total of three cycles. On day 3 following the final administration, primary tumors (final volume ~300–400 mm^3^) were surgically resected. Long-term survival and postoperative tumor recurrence were closely monitored thereafter.

For combination therapy with PD-L1 blockade, the mice received 200 μg/mouse of anti-PD-L1 antibody (B7-H1) every 6 days, alongside αHSA-proIL-12 administered every 3 days.

For the lung metastasis model, subcutaneous tumors were first established in mice. Then the mice received tail-vein injections of αHSA-proIL-12 on Days 0, 3, 6, and 9. Subsequently, 5 × 10^5^ B16F10 cells were injected intravenously to induce lung metastasis. Lungs were harvested on Day 20 for quantification of metastatic nodules.

### 4.8. Flow Cytometry Analysis

Tumor tissues were harvested and minced into small fragments and then digested with RPMI 1640 medium containing 0.5 mg/mL collagenase IV, 4 U/mL DNase I, and 10 mM HEPES at 37 °C for 45 min. Digested tumor cell suspensions were filtered through a 40 μm cell strainer into 50 mL centrifuge tubes. Immune cells were isolated by density gradient centrifugation. Prior to staining, the cells were incubated with a viability dye (Zombie UV387™ Fixable Viability Kit, BioLegend, San Diego, CA, USA) at 4 °C for 30 min, after which the Fc receptors were blocked to minimize nonspecific binding. After the unbound blocking antibodies were removed, the cells were stained with fluorochrome-conjugated antibodies against surface markers at 4 °C for 30 min in the dark. Fixation and permeabilization were performed using the eBioscience Foxp3 Transcription Factor Staining Buffer Set (Thermo Fisher Scientific, Waltham, MA, USA, 00-5521-00) in accordance with the manufacturer’s instructions. Following 1 h of fixation at 4 °C, the cells were washed with permeabilization buffer and stained with antibodies against intracellular markers overnight at 4 °C. On the following day, after the unbound antibodies were removed, the cells were resuspended in staining buffer and analyzed using a BD LSRFortessa flow cytometer (BD Biosciences, Milpitas, CA, USA). The specific antibody clones and conjugates used are as follows: CD45-BV510 (13/2.3), CD3-BV786 (17A2), CD8-PE (53-6.7), CD8-FITC (53-6.7), NK1.1-PE-Cy7 (PK136), CD4-BV605 (GK1.5), CD25-PerCP (3C7), Foxp3-AF647 (MF-14), CD62L-BV786 (MEL-14), CD3-APC-Cy7 (17A2), CD44-BV510 (IM-7), PD-1-APC (29F.1A12), Ki67-BV421 (16A8), CD11b-BUV396 (M1170), Gr1-BV421 (RB6-8C5), F4/80-PE (BM8), CD206-APC (C068C2), TNFα-BV650 (MP6-XT22), and Granzyme B-PE (QA18A28) (all purchased from BioLegend).

### 4.9. RNA-Seq

Untreated and treated tumor tissues were collected, snap-frozen in liquid nitrogen, and then stored at −80 °C to prevent RNA degradation. The tissues were disrupted by grinding or homogenization in liquid nitrogen grinding, and total RNA was extracted using TRIzol reagent or a commercial RNA extraction kit. Afterward, the RNA concentration was quantified using a Nanodrop or Qubit fluorometer, and RNA integrity was assessed by performing agarose gel electrophoresis or with a Bioanalyzer (with the RNA integrity number (RIN) recorded). Messenger RNA (mRNA) was enriched using oligo(dT) magnetic beads or ribosomal RNA (rRNA) was depleted, and the purified mRNA was fragmented into 200–300 bp fragments. Using the fragmented mRNA as the template, double-stranded cDNA was synthesized with reverse transcriptase. A cDNA library was constructed following the end repair, 3′-end adenylation, and ligation of sequencing adapters to the cDNA fragments. The library was amplified by PCR and purified; the library concentration was quantified using a Qubit instrument or quantitative PCR (qPCR), and the library size distribution was verified with a Bioanalyzer or TapeStation.

RNA-seq libraries were sequenced on the Illumina sequencing platform, and the resulting reads were aligned to the mouse mm10 reference genome. Raw sequencing reads underwent quality filtering using fastp according to the following criteria: reads contaminated with adapter sequences, containing more than 10% ambiguous N bases, or in which over 50% of bases exhibited a Phred quality score of ≤20 were excluded. Following filtration, clean reads with Q20 scores exceeding 97% and Q30 scores exceeding 94% were retained for downstream analyses. The sequencing depth per sample ranged from approximately 40 to 50 million clean reads. The clean reads were subsequently aligned to the mm10 genome using HISAT2, and transcript-level expression quantification was performed using StringTie. Differential gene expression analysis between groups with biological replicates was conducted using DESeq2, with thresholds |log2 (FC)| > 1 and adjusted *p* < 0.05. To elucidate the biological implications of the transcriptomic alterations, comprehensive functional enrichment analyses—including Gene Ontology (GO), Kyoto Encyclopedia of Genes and Genomes (KEGG), Reactome pathway enrichment, protein–protein interaction (PPI) network construction, and Gene Set Enrichment Analysis (GSEA)—were further carried out.

### 4.10. Statistical Analysis

The data are shown as the means ± SEM (standard error of the mean), unless stated otherwise. Statistical analyses were performed using GraphPad Prism statistical software. Two-tailed unpaired Student’s *t*-test, one-way ANOVA followed by Tukey’s multiple-comparison test, and the log-rank test were used for inter-group comparisons. Differences with *p* < 0.05 were considered statistically significant. The levels of significance were defined as: * *p* < 0.05, ** *p* < 0.01, *** *p* < 0.001, **** *p* < 0.0001 and ns (not significant). No experimental sample was excluded from analysis in all representative experiments. The detailed *p*-values for relevant comparisons are provided in Supplementary Tables S1–S11.

## 5. Conclusions

We successfully developed a tumor-conditional IL-12 prodrug named αHSA-proIL-12 to circumvent the inherent limitations of severe systemic toxicity and short circulatory half-life. This engineered molecule integrates an albumin-binding nanobody and a masking domain via an MMP-cleavable linker to achieve prolonged systemic circulation alongside precise activation within the tumor microenvironment. Our experimental findings confirm that this prodrug exhibits significantly reduced toxicity and superior antitumor efficacy which effectively widens the therapeutic window of IL-12. The prodrug promotes robust recruitment and activation of intratumoral CD8^+^ T cells and NK cells to successfully remodel the immunosuppressive microenvironment. Therapeutic evaluations across multiple tumor models demonstrate that αHSA-proIL-12 markedly suppresses tumor growth and extends overall survival. Furthermore, the platform shows enhanced therapeutic potential when used in combination with PD-L1 blockade or surgical intervention. In summary the αHSA-proIL-12 platform offers a safer and more potent strategy for cytokine-based cancer immunotherapy and possesses significant potential for clinical translation.

## Data Availability

The raw RNA-seq reads generated in this study have been deposited in the NCBI Sequence Read Archive (SRA) and will be made publicly available at the time of publication under the BioProject accession number PRJNA1515424. All other data that support the findings of this study are available from the corresponding author upon reasonable request.

## Supplementary Materials

The following supporting information can be downloaded at: https://www.mdpi.com/article/10.3390/ijms27177716/s1.
