# Supplementary material for: αHSA-proIL-12: A Tumor-Conditional IL-12 Prodrug with Reduced Systemic Toxicity and Potent Antitumor Activity
Source: Int J Mol Sci. 2026 Aug 28;27(17):7716. doi: 10.3390/ijms27177716 (PMC13566567; doi:10.3390/ijms27177716)
Supplement: Supplementary file 1 [file ijms-27-07716-s001.zip › ijms-4477662-supplementary.pdf]

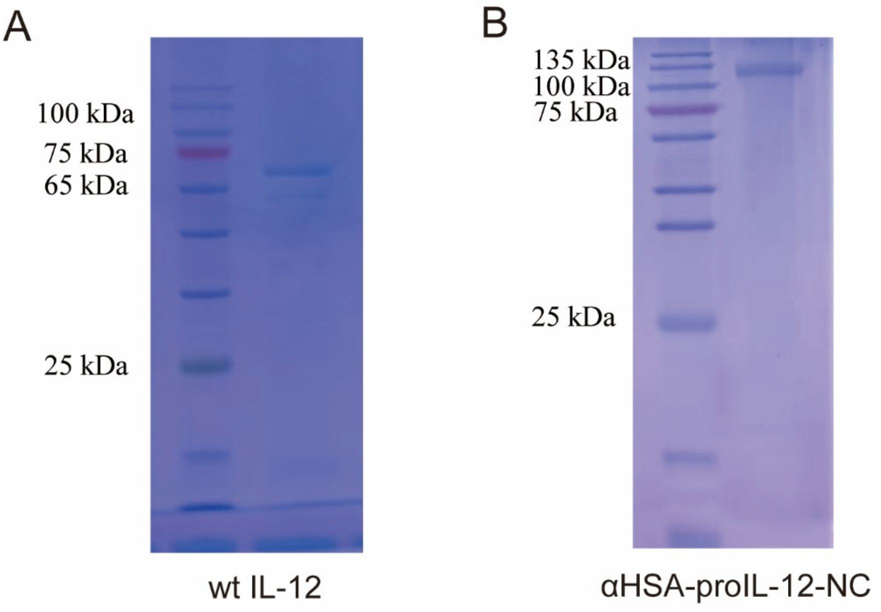

Supplementary Figure S1. Expression and purification of control molecules.

A. Sodium dodecyl sulfate–polyacrylamide gel electrophoresis (SDS–PAGE) analysis of wt IL-12 after expression and purification. The protein bands correspond to the heterodimeric structure of wt IL-12 (p40/p35 subunits), which is consistent with the predicted molecular weight.

B. SDS–PAGE analysis of the noncleavable protein variant (αHSA-proIL-12-NC) following purification via immobilized metal affinity chromatography. The gel was stained with Coomassie Brilliant Blue, and the expected molecular weight of the target protein is indicated.

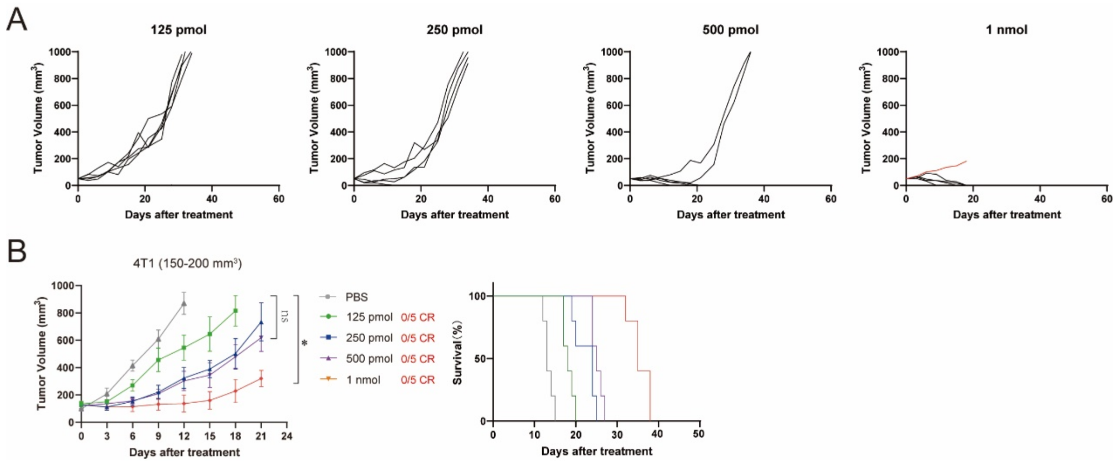

Supplementary Figure S2. αHSA-proIL12 treatment of 4T1 tumors.

A. Individual tumor growth curves of BALB/c mice bearing small 4T1 subcutaneous tumors (30–50 mm^3) following treatment with αHSA-proIL-12 or PBS (administered every 3 days for 7 doses). Tumor volumes were measured twice weekly using digital calipers.

B. Tumor growth curves (left panel) and Kaplan–Meier survival curves (right panel) of BALB/c mice bearing large 4T1 subcutaneous tumors (150–200 mm^3) treated with αHSA-proIL-12 or PBS. Survival was monitored until the experimental endpoint (tumor volume ≥1500 mm^3 or meeting the humane endpoint criteria).

The data are shown as the means ± SEMs. Statistical significance was determined using one-way analysis of variance (ANOVA) with Tukey's multiple comparisons test. *$p < 0.05$, **$p < 0.01$, ***$p < 0.001$, ****$p < 0.0001$, and ns = not significant.

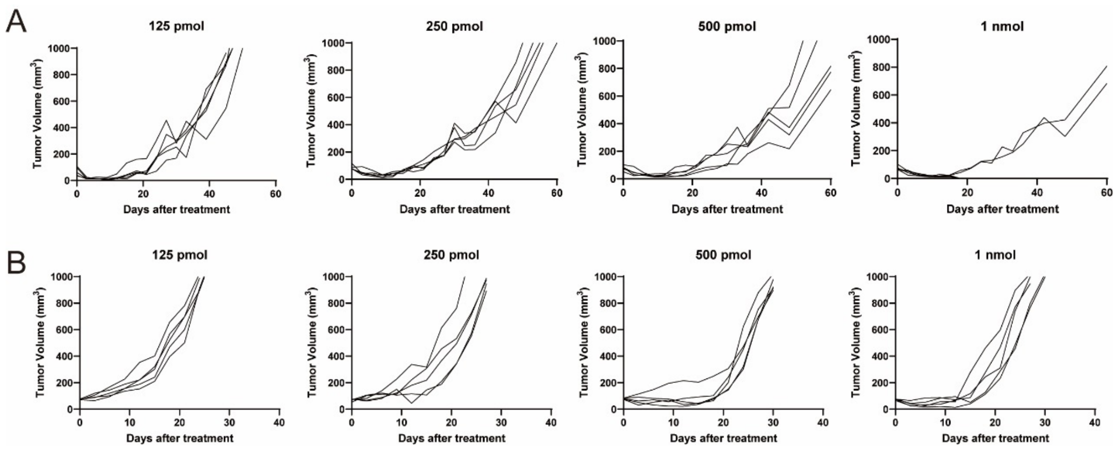

Supplementary Figure S3. Therapeutic experiments in other immunologically "cold" tumor models.

A. Individual tumor growth curves of C57BL/6N mice bearing subcutaneous Panc02 tumors (~100 mm^3) treated with αHSA-proIL12 or PBS (dosing schedule: every 3 days for 7 doses). Tumor volumes were assessed twice weekly.

B. Individual tumor growth curves of C57BL/6N mice bearing subcutaneous B16F10 tumors (~100 mm^3) following αHSA-proIL12 or PBS treatment (administered every 3 days for 7 doses).

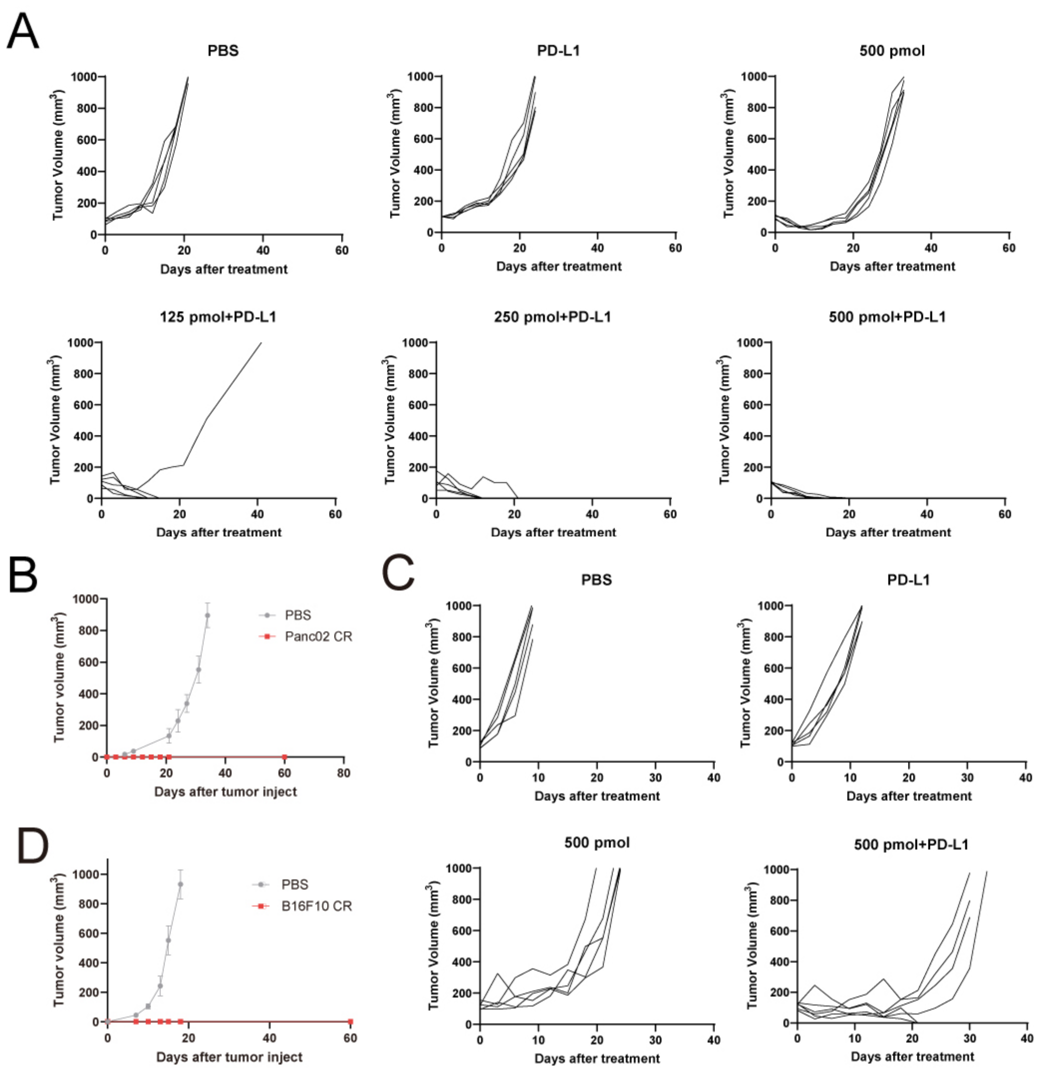

Supplementary Figure S4. Combination therapy consisting of αHSA-proIL12 with a PD-L1 antibody in immunologically "cold" tumors.

A. Individual tumor growth curves of C57BL/6N mice bearing subcutaneous Panc02 tumors (~100 mm^3) treated with αHSA-proIL-12 + anti-PD-L1 antibody, αHSA-proIL-12, anti-PD-L1 antibody, or PBS.

B. Tumor growth curves of Panc02 tumor-free mice (cured by the combination therapy) following rechallenge with 5×10^5 Panc02 cells (subcutaneous injection).

C. Individual tumor growth curves of C57BL/6N mice bearing subcutaneous B16F10 tumors (100 mm^3) treated with αHSA-proIL-12 + anti-PD-L1 antibody, monotherapy, or PBS.

D. Tumor growth curves of B16F10 tumor-free mice (cured by the combination therapy) following reinoculation with 5×10^5 B16F10 cells (subcutaneous injection).

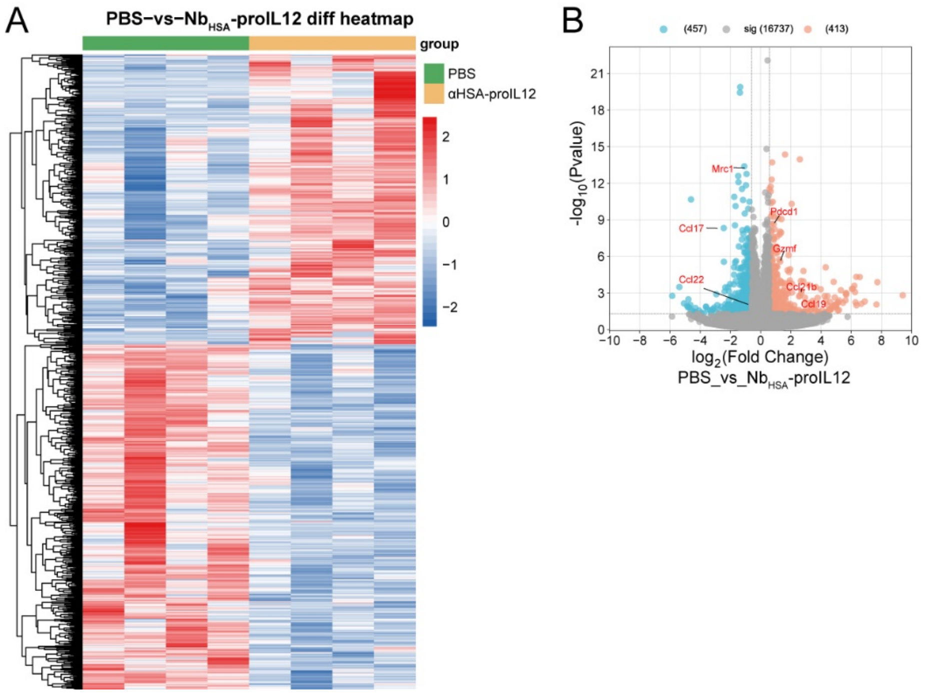

Supplementary Figure S5. Summary of bulk RNA-seq data from the MC38 tumor model.

A. Hierarchical clustering heatmap of all differentially expressed genes (DEGs; adjusted $p < 0.05$, $|\log_2(\text{fold change})| \geq 1$) identified in MC38 tumor tissues from αHSA-proIL-12-treated mice compared with those from PBS control mice. The color intensity represents the z score-normalized gene expression levels (blue: downregulated; red: upregulated).

B. Volcano plot showing all genes detected in the bulk RNA-seq dataset. The x-axis denotes the $\log_2$(fold change) (αHSA-proIL-12 vs. PBS), and the y-axis denotes the $\log_{10}$(p-value). Significantly differentially expressed genes ($p < 0.05$) are highlighted in red (upregulated) or green (downregulated); key immune-related genes (e.g., *Ifng* and *Cxcl9*) are labeled.

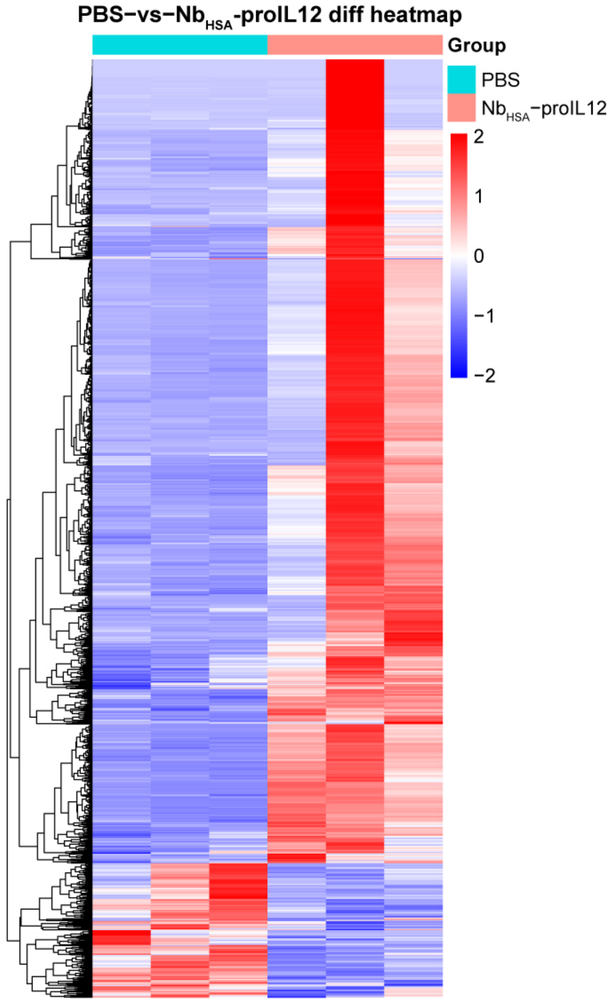

Supplementary Figure S6. Bulk RNA-seq analysis of the B16F10 tumor model. Hierarchical clustering heatmap of all differentially expressed genes (DEGs; $p < 0.05$, $|\log_2(\text{fold change})| \geq 1$) in B16F10 tumor tissues from αHSA-proIL-12-treated mice compared with those from PBS control mice. Rows represent individual genes, and columns represent biological replicates; the color intensity indicates z score-normalized expression (blue: low; red: high).

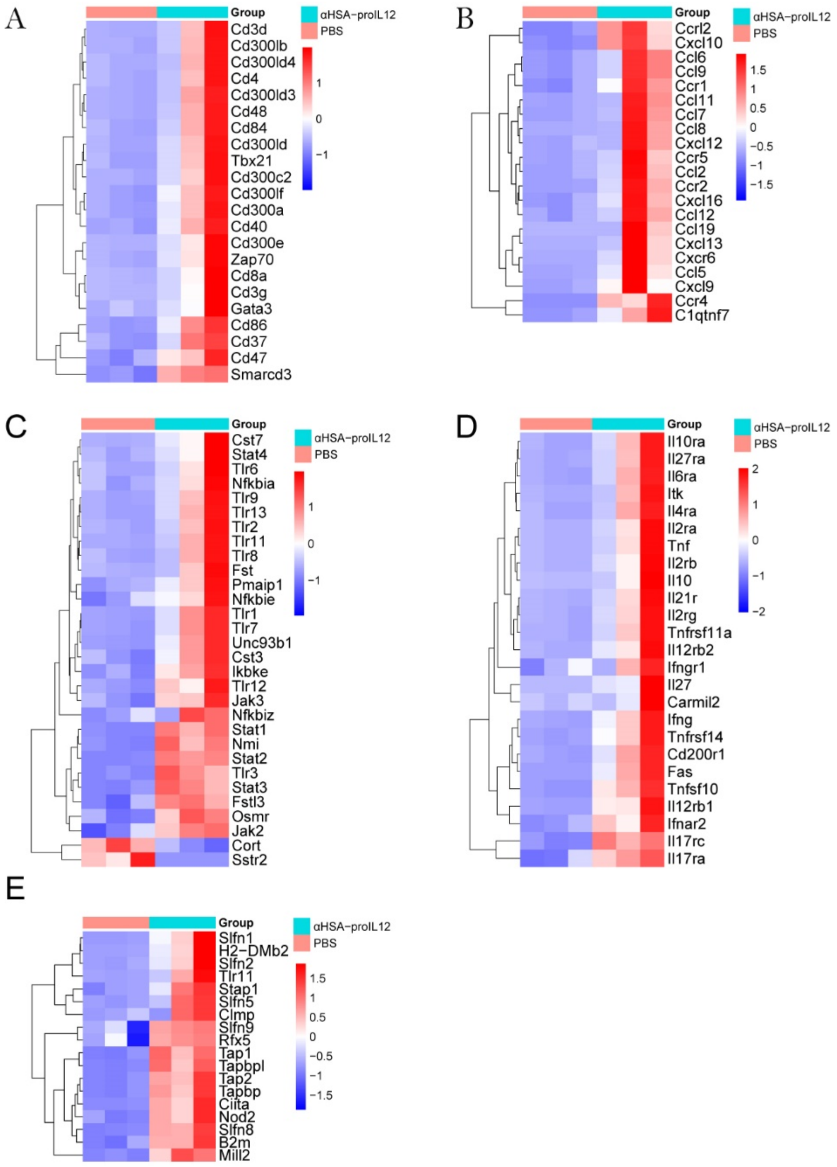

Supplementary Figure S7. Heatmap of differentially expressed genes identified in the RNA-seq data from B16F10 tumors.

A. Genes linked to immune cell function (e.g., activation, proliferation, and cytotoxicity).

B. Genes encoding chemokines and chemokine receptors.

C. Genes involved in canonical immune signaling pathways (e.g., JAK–STAT and NF-κB signaling).

D. Genes encoding cytokines and their cognate receptors.

E. Genes associated with antigen processing and presentation (e.g., MHC molecules and antigen transporters).

For all panels, the x-axis = $\log_2$(fold change); y-axis = $-\log_{10}$(p value); red/green dots = significantly upregulated/downregulated genes ($p < 0.05$).

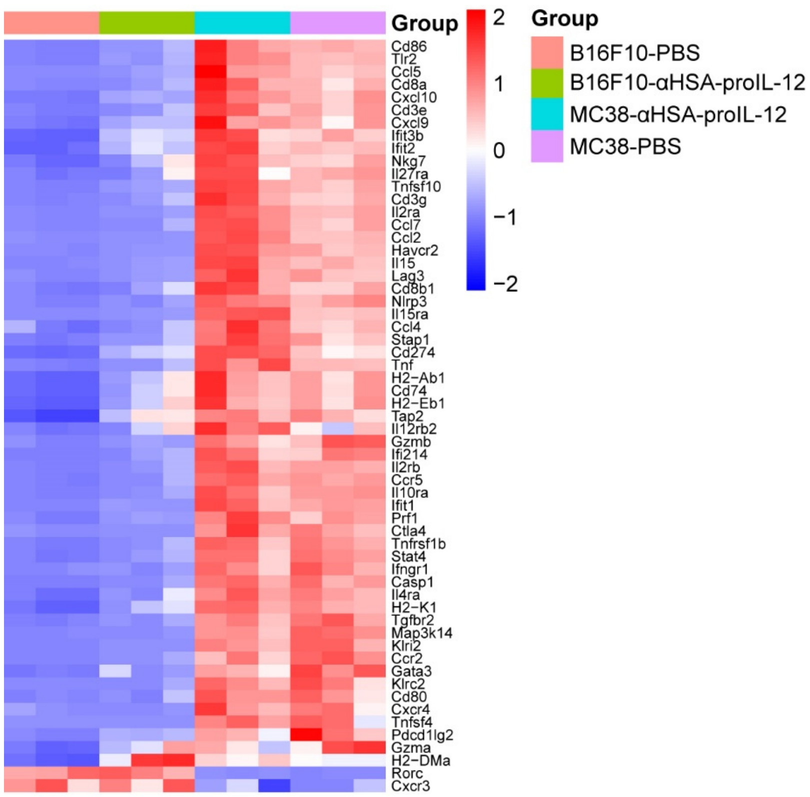

Supplementary Figure S8. Heatmap of immune-related gene expression profiles in B16F10 and MC38 tumors treated with αHSA-proIL-12 or PBS.

The heatmap displays the normalized expression levels of key immune-related genes across the four experimental groups: B16F10-PBS, B16F10- , MC38-αHSA-proIL-12, and MC38-PBS. The color scale represents the $\log_2$-transformed fold change, with red indicating upregulation and blue indicating downregulation.

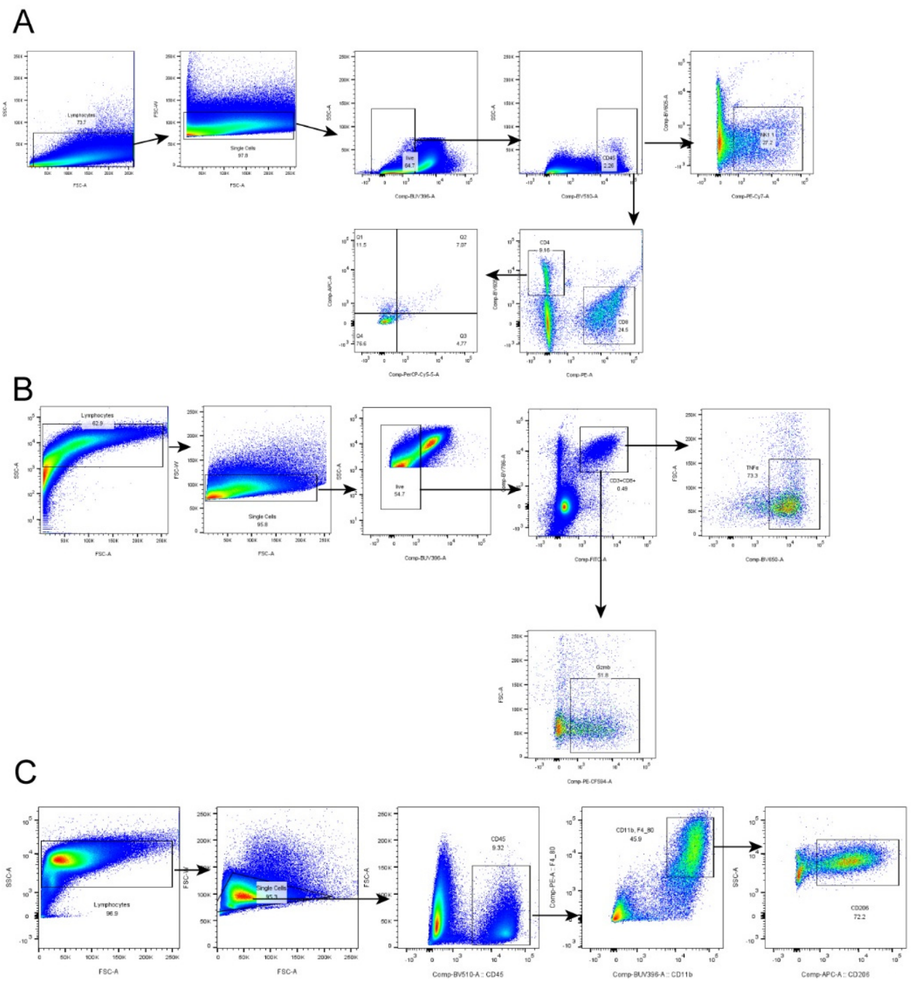

Supplementary Figure S9. Flow cytometry gating strategies

A. Gating strategy for $CD8^+$ T cells, $CD4^+$ T cells, NK cells, and Treg cells.

B. Gating strategy for cytokine-secreting $CD8^+$ T cells.

C. Gating strategy for macrophages and their subsets.

In view of the prominent species specificity of IL-12 receptor signaling, the therapeutic outcomes derived from wild-type C57BL/6 mice may not reflect human physiological responses. To overcome this limitation, we assessed the antitumor activity of αHSA-prohIL-12, a human IL-12 variant of αHSA-proIL-12, in humanized mice engineered to express human IL-12Rβ1 and IL-12Rβ2. In this study, the mice were subcutaneously injected with MC38 tumor cells. Upon reaching a volume of 100–150 mm^3, the mice were randomized and intravenously administered 250 pmol/mouse αHSA-prohIL-12 via the tail vein every 3 days for three doses. The tumor dimensions and body weights were monitored at regular intervals to evaluate therapeutic efficacy and systemic tolerability.

As depicted in Figure S10, PBS-treated control tumors progressed aggressively, surpassing 1000 mm^3 in all animals by day 18. Conversely, αHSA-prohIL-12 elicited marked tumor inhibition from the initial dose onward, with sustained regression observed following the third injection, culminating in complete tumor eradication in all treated mice. Throughout the treatment course, the body weights remained unaltered, confirming the favorable systemic safety profile of αHSA-prohIL-12 at the administered dose.

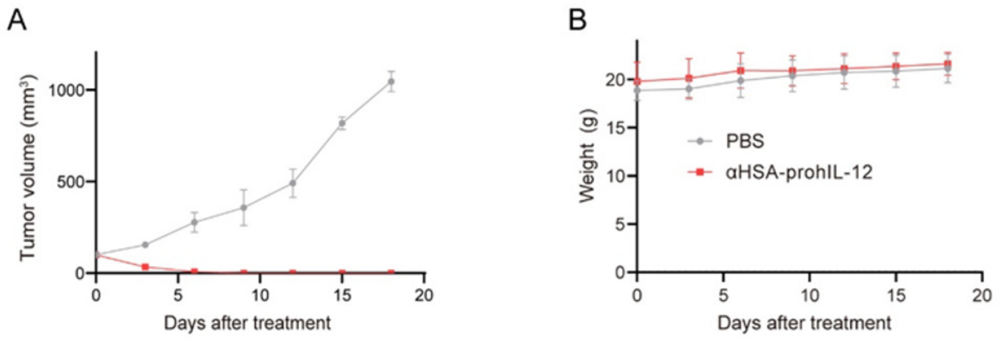

Supplementary Figure S10. Human αHSA-prohIL-12 mediates robust anti-tumor activity against MC38 tumors in IL-12 receptor humanized knock-in mice (B-hIL12RB1/hIL12RB2).

Mice were dosed with recombinant human αHSA-prohIL-12 every three days.

A. Tumor growth curves of B-hIL12RB1/hIL12RB2 mice bearing subcutaneous MC38 tumors (~100 mm^3) treated with αHSA-prohIL-12 (human IL-12 variant of αHSA-proIL-12) or PBS. The mice were dosed once every three days.

B. Mouse body weight kinetics during therapy.

Supplementary Table S1. Statistical comparison of tumor volume between groups (Figure 2A)

Comparison (Day 15)	Mean difference	95% CI	P value
PBS vs 4 pmol	508.0	356.3 to 659.7	<0.0001
PBS vs 12 pmol	684.6	532.9 to 836.3	<0.0001
PBS vs 36 pmol	775.6	623.9 to 927.3	<0.0001
PBS vs 108 pmol	819.6	667.9 to 971.3	<0.0001
PBS vs 216 pmol	810	658.3 to 961.7	<0.0001

Supplementary Table S2. Statistical comparison of tumor volume between groups (Figure 2B)

Comparison (Day 18)	Mean difference	95% CI	P value
PBS vs 125 pmol	153.6	-108.5 to 415.7	0.3632
PBS vs 250 pmol	556.8	294.7 to 818.9	<0.0001
PBS vs 500 pmol	806.6	544.5 to 1069	<0.0001
PBS vs 1 nmol	1037	774.9 to 1299	<0.0001

Supplementary Table S3. Statistical comparison of ALT and AST between groups (Figure 2E)

Comparison	Mean difference	95% CI	P value
AST<br>PBS vs wt IL-12	-37.62	-70.53 to -4.707	0.0299
AST<br>PBS vs αHSA-proIL-12	-2.055	-34.97 to 30.86	0.9769
ALT<br>PBS vs wt IL-12	-73.95	-144.3 to -3.639	0.0414
ALT<br>PBS vs αHSA-proIL-12	-7.709	-78.02 to 62.61	0.9316

Supplementary Table S4. Statistical comparison of tumor volume between groups (Figure 3A)

Comparison (Day 18)	Mean difference	95% CI	P value
PBS vs 54 pmol	373.6	141.8 to 605.4	0.0024
PBS vs 108 pmol	695.6	463.8 to 927.4	<0.0001

PBS vs 216 pmol	779.6	547.8 to 1011	<0.0001

Supplementary Table S5. Statistical comparison of Figure 3F.

Comparison	Mean difference	95% CI	P value
TEM<br>PBS vs αHSA-proIL-12	-5.74	-7.073 to -4.407	<0.0001
TCM<br>PBS vs αHSA-proIL-12	-10.12	-16.04 to -4.196	0.0043

Supplementary Table S6. Statistical comparison of tumor volume between groups (Figure 4B)

Comparison (Day 15)	Mean difference	95% CI	P value
PBS vs NC 1 nmol	561	453.5 to 668.5	<0.0001
PBS vs 125 pmol	647.4	539.9 to 754.9	<0.0001
PBS vs 250 pmol	764.4	656.9 to 871.9	<0.0001
PBS vs 500 pmol	815.6	708.1 to 923.1	<0.0001
PBS vs 1 nmol	812.8	705.3 to 920.3	<0.0001

Supplementary Table S7. Statistical comparison of tumor volume between groups (Figure 5B)

Comparison (Day 21)	Mean difference	95% CI	P value
PBS vs NC 1nmol	782.2	667.4 to 897.0	<0.0001
PBS vs 125 pmol	913.6	798.8 to 1028	<0.0001
PBS vs 250 pmol	894.0	779.2 to 1009	<0.0001
PBS vs 500 pmol	916.0	801.2 to 1031	<0.0001
PBS vs 1 nmol	962.0	847.2 to 1077	<0.0001

Supplementary Table S8. Statistical comparison of tumor volume between groups (Figure 5F)

Comparison (Day 15)	Mean difference	95% CI	P value
PBS vs 125 pmol	695.6	567.6 to 823.6	<0.0001
PBS vs 250 pmol	771.0	643.0 to 899.0	<0.0001
PBS vs 500 pmol	906.0	778.0 to 1034	<0.0001
PBS vs 1 nmol	875.0	747.0 to 1003	<0.0001

Supplementary Table S9. Statistical comparison of metastatic foci between groups (Figure 5K)

Comparison	Mean difference	95% CI	P value
PBS vs αHSA-proIL-12	278.8	15.88 to 541.7	0.0402

Supplementary Table S10. Statistical comparison of tumor volume between groups (Figure 6B)

Comparison (Day 24)	Mean difference	95% CI	P value
PD-L1 vs 500 pmol	652.4	474.5 to 830.3	<0.0001
PD-L1 vs 125 pmol+PD-L1	826.0	648.1 to 1004	<0.0001
PD-L1 vs 250 pmol+PD-L1	879.4	701.5 to 1057	<0.0001
PD-L1 vs 500 pmol+PD-L1	899.0	721.1 to 1077	<0.0001

Supplementary Table S11. Statistical comparison of tumor volume between groups (Figure 6E)

Comparison	Mean difference	95% CI	P value
PD-L1 vs 500 pmol αHSA-proIL-12 (Day 12)	720.4	632.8 to 808.0	<0.0001
PD-L1 vs 500 pmol αHSA-proIL-12+PD-L1 (Day 12)	854.7	770.8 to 938.6	<0.0001
500 pmol αHSA-proIL-12 vs 500 pmol αHSA-proIL-12+PD-L1 (Day 21)	570.8	272.9 to 868.6	0.0006
